# Natural Polysaccharide-Based Biomaterials for Skin Wound Healing: Immunomodulatory Mechanisms and Macrophage M2 Polarization

**DOI:** 10.3390/gels12090794

**Published:** 2026-09-01

**Authors:** Zhe Huang, Yubo Di, Luyao Wen, Xing He, Weiwei Zhang, Qingcong Wei

**Affiliations:** Key Laboratory of Green Chemical Media and Reactions, Ministry of Education, Henan Engineering Research Centre of Chiral Hydroxyl Pharmaceutical, School of Chemistry and Chemical Engineering, Henan Normal University, Xinxiang 453007, China; 17527051052@163.com (Z.H.); yubodi2026@163.com (Y.D.); wenluyao@stu.htu.edu.cn (L.W.); hexing@htu.edu.cn (X.H.)

**Keywords:** natural polysaccharide-based biomaterials, macrophage polarization, M2 phenotype, wound healing, immunomodulation

## Abstract

Efficient cutaneous wound healing relies on the phenotypic transition of macrophages toward an anti-inflammatory, pro-reparative M2-like state. Non-healing chronic wounds are pathologically characterized by the breakdown of this polarization balance. In this review, we synthesize recent research on biomaterials fabricated from naturally occurring polysaccharides with intrinsic immunomodulatory activity, mainly represented by hydrogels that modulate macrophage phenotypic transitions. We first dissect the immune microenvironment of wound healing and elaborate on the core regulatory networks governing M1/M2 polarization, with a particular focus on signaling pathways and metabolic reprogramming. On this basis, we classify pro-M2 natural polysaccharides into mannose-containing and mannose-free categories according to their core structural motifs that mediate immunomodulatory activity, and detail their molecular mechanisms, including pattern recognition receptor engagement (MR, Dectin-1, CD44, etc.) and downstream signaling cascades (STAT6, PI3K/Akt, NF-κB, etc.). Representative polysaccharides such as konjac glucomannan (KGM), *Ganoderma lucidum* polysaccharide (GLP), chitosan (CS) and hyaluronic acid (HA) are discussed with a clarified structure–activity relationship (SAR). Finally, we highlight emerging design strategies for multi-functional immunomodulatory hydrogels, including mechano-biochemical coupling platforms and spatiotemporally controlled delivery systems, and analyze ongoing controversies and translational bottlenecks in this field. The relationship between material structure and function enables the rational design of purpose-built polysaccharide dressings that regulate immunity. This review highlights these materials as promising preclinical platforms for chronic wound management, although their clinical translation requires further validation.

## 1. Introduction

Skin protects underlying tissues from physical damage, bacteria, UV light and extreme temperatures. It is the body’s largest organ and forms our main outer defense. Wounds, burns, surgical incisions, and long-term illnesses such as diabetes can damage the structure of the skin. When such damage occurs, the skin can no longer act as a protective barrier. The body subsequently initiates a coordinated series of biological responses to repair the injured skin surface, a process collectively termed wound healing. All stages of skin repair overlap with one another and follow strict biological control mechanisms. The complete biological sequence of skin lesion repair consists of four distinct core phases. These sequential stages cover hemostasis, inflammation, cell proliferation, and tissue remodeling respectively [1,2,3].

Upon entering the inflammatory stage, wounds are rapidly infiltrated by neutrophils and macrophages. These immune cells cooperate to eradicate invading pathogens and clear necrotic tissue debris. Macrophages serve as pivotal regulators in the healing process due to their high plasticity and functional diversity [4].

Macrophage polarization exists along a continuous functional spectrum rather than as discrete binary states. Nonetheless, for narrative clarity, the M1/M2 nomenclature is widely adopted to describe the pro-inflammatory and anti-inflammatory extremes of this spectrum, respectively [5]. Classically activated M1 macrophages trigger inflammation and release TNF-α, IL-6 and other pro-inflammatory molecules to eliminate invading pathogens. However, while persistent M1 macrophage accumulation undoubtedly exacerbates local tissue damage, it is equally imperative to recognize that the early inflammatory response dominated by M1 cells is indispensable for effective pathogen clearance and necrotic debris removal [6]. Conversely, although M2 macrophages exert anti-inflammatory and pro-reparative functions, premature or excessive M2 polarization can drive aberrant TGF-β signaling and predispose the wound to subsequent fibrosis. Therefore, optimal healing outcomes do not depend on unidirectional M2 amplification, but rather on the precise spatiotemporal regulation of the M1-to-M2 phenotypic transition, which must avoid both protracted inflammation and untimely immunosuppression [7]. It should be noted, however, that the commonly used M2-associated markers, particularly arginase-1(Arg-1) and CD206, exhibit differential expression profiles between murine and human macrophages. While these markers are robustly validated in mouse models, their translational applicability to human wound healing requires cautious interpretation, as human macrophages display distinct metabolic and transcriptional regulation of these molecules under equivalent polarizing conditions. Throughout this review, evidence derived from murine studies is explicitly distinguished from human-derived data, and marker interpretation is contextualized accordingly. Alternatively activated M2 macrophages predominate in the later stages of inflammation, producing IL-10, TGF-β and other multifunctional substances. These substances lower inflammation and help tissue heal by promoting new blood vessels, enhancing fibroblast proliferation, stimulating collagen synthesis and facilitating tissue remodeling [4].

Normal skin healing maintains a balanced cycle of inflammation and tissue regeneration; however, conditions such as diabetes and venous insufficiency disrupt this equilibrium. This breakdown results in three interconnected pathologies: unresolved inflammation, defective macrophage M2 polarization, and wounds arrested in a protracted inflammatory state [8,9]. Diabetic wounds are situated in a hyperglycemic microenvironment that promotes the persistent accumulation of advanced glycosylation end products (AGEs). A large body of research shows these accumulated substances activate inflammatory signaling pathways, which leads to an overgrowth of M1 macrophages and reduces the normal function of M2 cells [6,9]. Non-healing chronic wounds are optimally managed by therapeutic strategies centered on two core approaches. One method guides macrophages to switch their cell type from M1 to M2, while the other reconstructs a wound microenvironment filled with repair-friendly immune signals [6,10].

Among the diverse polysaccharide-based biomaterial platforms, hydrogels have emerged as the most extensively investigated archetype. Hydrogels can generate a desirable moist microenvironment for wound repair. Their porous network architecture, large water loading capacity, mild biocompatibility and alterable physicochemical traits jointly support this function [11]. Nevertheless, non-hydrogel formulations—including electrospun nanofibrous membrane (ENM), porous sponges, and microneedle arrays—offer distinct mechanobiological or delivery advantages that complement hydrogel-based strategies, as discussed in the following sections.

In particular, natural polysaccharides from specific sources are not only ideal for the construction of the hydrogel skeleton, but also have excellent immunomodulatory activity, which can actively intervene in the wound microenvironment as “biological response modifiers” [12]. Upon binding to pattern recognition receptors on macrophage outer membranes, specific polysaccharides can initiate downstream signaling cascades that have been frequently reported to be associated with guiding macrophages toward an M2-like functional state [13,14]. Nevertheless, it must be emphasized that such receptor engagement does not automatically equate to deterministic M2 polarization, as the ultimate biological outcome is profoundly modulated by multiple physicochemical variables, including molecular weight, branching degree, purity, administered dose, macrophage source, and the prevailing inflammatory milieu [15,16]. More specifically, parameters such as the degree of acetylation or deacetylation, residual protein or polyphenolic impurities derived from raw material extraction, and trace endotoxin contamination have all been demonstrated to profoundly confound macrophage responses in polysaccharide studies. These variables are explicitly addressed in the structure–activity relationship analysis presented in subsequent sections. In practical applications, polysaccharide-based dressings are often fabricated as composite systems loaded with supplementary active agents such as antimicrobials, metallic nanoparticles, or growth factors. In such cases, the polysaccharide may fulfill a triple functional role: as an immunomodulatory entity that governs macrophage phenotypes, as a structural scaffold providing mechanical support and moisture balance, or as a delivery vehicle for controlled release of therapeutic cargo. Disambiguating these distinct roles is critical to avoid overattributing therapeutic efficacy solely to macrophage polarization when other bioactive components are present.

**Figure 1:**
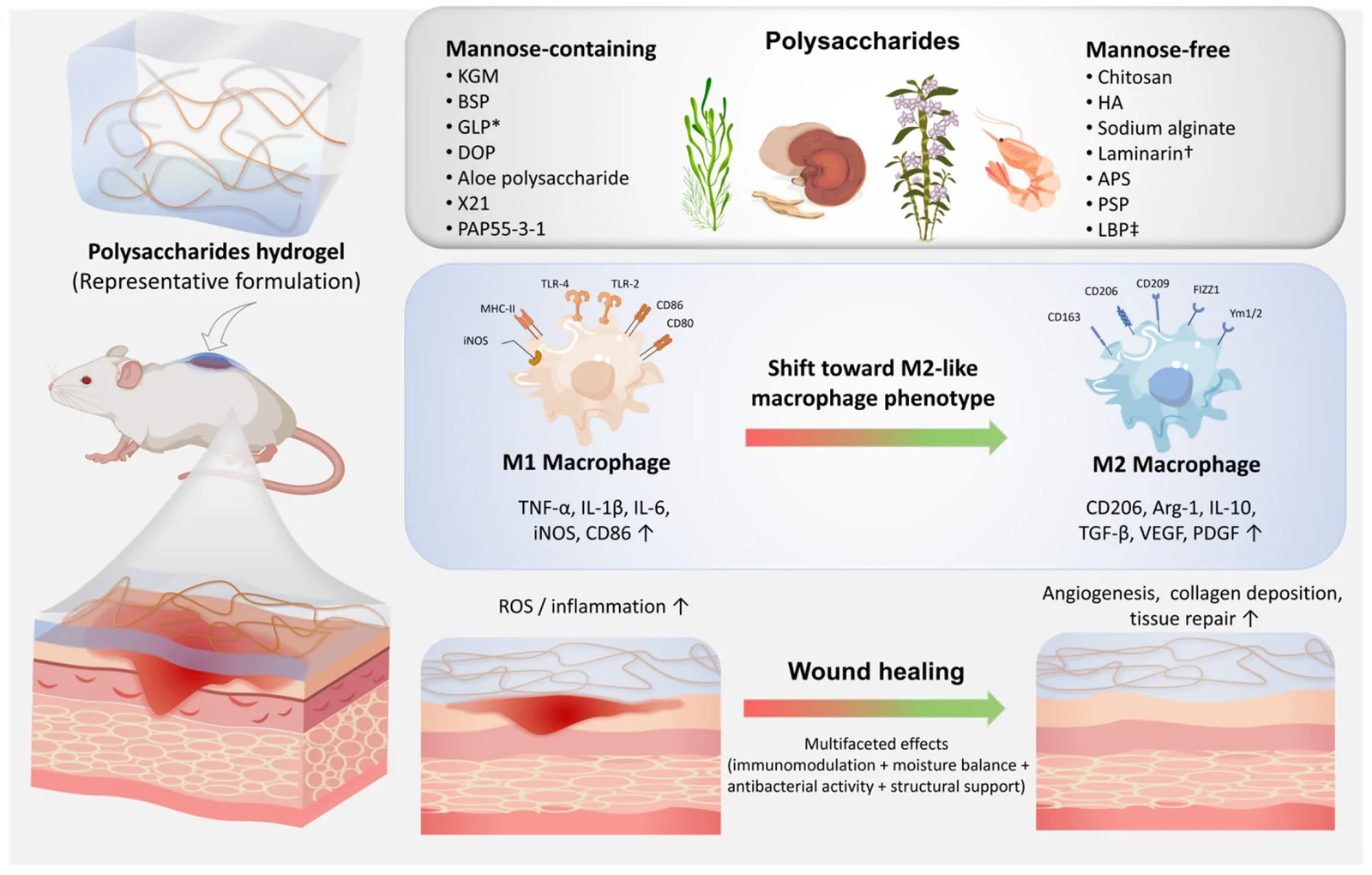
Schematic overview of natural polysaccharide-based biomaterials promoting skin wound healing via macrophage immunomodulation. Polysaccharide hydrogels are displayed as the representative core formulation of all polysaccharide dressing platforms. Natural bioactive polysaccharides are grouped into mannose-containing and mannose-free categories according to the operational structural–functional criterion detailed in Section 3.1. Representative mannose-containing varieties include KGM, Bletilla striata polysaccharide (BSP), Dendrobium officinale polysaccharide (DOP), aloe polysaccharide, Schizophyllum commune polysaccharide, and GLP*. Mannose-free candidates include CS, HA, sodium alginate (primarily structural scaffold with negligible intrinsic immunomodulatory activity), Astragalus polysaccharide (APS), Hylocereus undatus stem polysaccharide (PSP), laminarin†, Lycium barbarum polysaccharide (LBP)‡, Anoectochilus xingrenensis polysaccharide (X21), and Periplaneta americana polysaccharide (PAP55-3-1). These polysaccharides can be fabricated into diverse biomaterial platforms, including hydrogels, films, sponges, aerogels, nanofibers, and microneedles. The diagram displays a generalized conceptual pathway rather than universally validated mechanisms for all listed biomaterials: certain polysaccharides can bind pattern recognition receptors on pro-inflammatory macrophages to tune downstream signaling cascades and drive an M2-like anti-inflammatory phenotypic shift. Notably, direct receptor-polysaccharide binding has only been experimentally verified for a limited subset of these materials; the immunomodulatory mechanisms of the remaining polysaccharides are merely inferred from cellular phenotypic changes and gene expression profiles (see Section 3). For composite dressings loaded with antimicrobials, nanoparticles or growth factors, the independent macrophage-regulating effect of polysaccharides must be distinguished from synergistic effects of supplementary bioactive ingredients (see main text). * GLP is retained in the mannose-containing group as a mechanistic borderline case because, despite its mannose content of 10–20%, it simultaneously interacts with both the mannose receptor (MR) and Dectin-1. † Laminarin is classified as mannose-free based on its glucose-dominant backbone; its immunomodulatory function relies on Dectin-1-mediated β-glucan recognition rather than mannose residue binding (see Section 3.1 criteria). ‡ LBP contains only 5–10 mol% mannose and exerts M2-polarizing effects independent of mannose residues and MR ligation. Its core functional motifs are highly branched arabinogalactan side chains, which suppress NF-κB/MAPK pro-inflammatory signaling pathways.

Existing reviews on polysaccharide wound dressings are mostly categorized by material source or dosage form, and lack systematic induction of the SAR between polysaccharide structural motifs and macrophage immunomodulatory activity. To address this gap in the literature, the present review discusses two core subjects: polysaccharide-based biomaterials—with a primary focus on hydrogels but also encompassing films, sponges, aerogels, nanofibers, and microneedles—for skin wound healing, and these bioactive polysaccharides themselves, which selectively drive macrophages to adopt anti-inflammatory, repair-facilitating M2 features. We first provide an in-depth analysis of the wound immune microenvironment and the core regulatory network of M1/M2 polarization. We then categorize pro-M2 polysaccharides based on their core functional structural motifs (mannose-containing vs. mannose-free), systematically review their immunomodulatory mechanisms and dressing construction strategies, and summarize the general rules of SAR. This review concludes by examining the latest biomaterial design strategies, along with the challenges hindering clinical translation. Collectively, this analysis provides a theoretical framework to guide the rational design of wound dressings capable of modulating the local immune response (Figure 1).

### 1.1. Significance and Novelty of This Review

To our knowledge, this is the first review to systematically integrate natural polysaccharide structural motifs with macrophage M2-polarizing mechanisms in the context of skin wound healing. Unlike existing reviews that categorize polysaccharide dressings by material source or dosage form, the present work: (i) establishes a receptor-centric mechanistic framework linking polysaccharide structure to immunomodulatory activity; (ii) critically distinguishes direct receptor-binding evidence from indirect pathway associations, thereby clarifying the current depth of mechanistic understanding; (iii) stratifies the functional roles of polysaccharides within composite systems as immunomodulatory agents, structural scaffolds, or drug carriers; and (iv) integrates the temporal dimension of macrophage reprogramming into material design considerations. By converging material science with macrophage biology, this review aims to provide a rational design roadmap for next-generation immunomodulatory wound dressings.

### 1.2. Scope and Literature Selection

This narrative review systematically summarizes the immunomodulatory mechanisms of natural polysaccharide-based biomaterials for skin wound healing, with a particular focus on macrophage M2 polarization. Hydrogels are adopted as the core, most extensively studied representative biomaterial form throughout this work, while complementary formulations including films, sponges, aerogels, nanofibers, and microneedles are also discussed as auxiliary expansion to build a complete framework of polysaccharide wound dressings. Relevant publications were identified primarily through PubMed, and additional records were retrieved from Web of Science, Scopus, and Google Scholar to ensure comprehensive coverage of both the materials science and immunological literature. Further relevant studies were obtained by manually screening the reference lists of selected articles and key reviews.

The literature search employed Boolean combinations of three keyword groups: (i) polysaccharide-related terms (e.g., “polysaccharide,” “glucomannan,” “chitosan,” “hyaluronic acid,” “alginate,” “laminarin,” “natural polysaccharide hydrogel”); (ii) immunomodulation and macrophage-related terms (“macrophage polarization,” “M1/M2 macrophage,” “mannose receptor,” “Dectin-1,” “STAT6,” “PI3K/Akt,” “NF-κB”); and (iii) wound healing-related terms (“wound healing,” “skin regeneration,” “chronic wound,” “diabetic ulcer,” “wound dressing”). Keywords from the three groups were combined using the AND operator across groups and the OR operator within each group, and were searched in titles, abstracts, and keywords.

The search covered publications from January 2000 to 6 August 2026, with the final search conducted on 6 August 2026. This timeframe captures both foundational studies in polysaccharide immunology and recent advances in biomaterial-based wound healing. No language restrictions were imposed at the initial search stage; however, non-English articles were included only when a complete English abstract or full-text translation was available.

Studies were included if they met one or more of the following criteria: (i) elucidated molecular mechanisms by which natural polysaccharides regulate macrophage polarization via pattern recognition receptors or downstream signaling; (ii) described the design and functional evaluation of polysaccharide-based hydrogels, films, sponges, aerogels, nanofibers, or microneedles for skin wound healing with experimental assessment of macrophage phenotype; or (iii) provided comparative data on polysaccharide structural features and their correlation with immunomodulatory potency. Non-cutaneous studies (e.g., intestinal inflammation, spinal cord injury, rheumatoid arthritis) were selectively included only when they provided direct mechanistic evidence for receptor–ligand interactions or signaling pathways conserved across macrophage populations, and were explicitly treated as mechanistic corroboration rather than efficacy evidence for cutaneous repair.

Studies were excluded if they: (i) were conference abstracts, editorials, or opinion pieces without original data; (ii) lacked sufficient methodological detail for critical evaluation; (iii) reported only material characterization without biological assessment relevant to macrophage polarization; (iv) focused on non-polysaccharide-based or non-natural polysaccharide materials outside the review scope; or (v) were duplicative publications without novel contributions. Priority was given to peer-reviewed original articles combining material characterization with in vitro macrophage phenotyping and/or in vivo wound-healing endpoints. Studies providing direct evidence of polysaccharide-receptor binding were distinguished from those reporting only indirect pathway associations. For composite systems containing antibiotics, nanoparticles, or growth factors, the specific contribution of the polysaccharide to M2 polarization was critically evaluated. The selected examples are illustrative rather than exhaustive.

Delineation of non-cutaneous evidence: throughout Section 3 and Section 4, non-cutaneous studies (e.g., intestinal inflammation, spinal cord injury, rheumatoid arthritis, myocardial infarction) are selectively cited solely to provide mechanistic corroboration for receptor–ligand interactions or intracellular signaling pathways that are conserved across macrophage populations. These citations are explicitly distinguished from cutaneous efficacy data and should not be interpreted as direct evidence of skin wound-healing outcomes. In the main text, such instances are parenthetically tagged as (mechanistic corroboration only) to alert readers that the experimental context differs from cutaneous repair.

## 2. Immune Microenvironment and Macrophage Polarization Regulation in Wound Healing

### 2.1. Stages of Wound Healing and the Dynamic Evolution of Macrophages

Skin repair unfolds across four intermingled, well-regulated phases: hemostasis, inflammation, proliferation and remodeling. This dynamic biological process is orchestrated through continuous interactions among diverse cell populations, signaling pathways, and extracellular matrix (ECM) components [1,3,6,15].

Upon wounding, the hemostasis phase is initiated by platelet aggregation and clot formation. These active platelets release PDGF, TGF-β and several chemokines, which in turn recruit inflammatory cells to the wound site. When wounds enter the inflammatory repair stage, immune cells invade injured tissue in a fixed sequence, with neutrophils migrating ahead of macrophages. This ordered infiltration characterizes the inflammatory phase. As the earliest immune cells arriving at wound lesions, neutrophils eliminate necrotic tissue and microorganisms by engulfing pathogens, releasing granular cargo, and generating neutrophil extracellular traps [17,18,19]. Subsequently, circulating monocytes are recruited and differentiate into macrophages [20,21].

**Figure 2:**
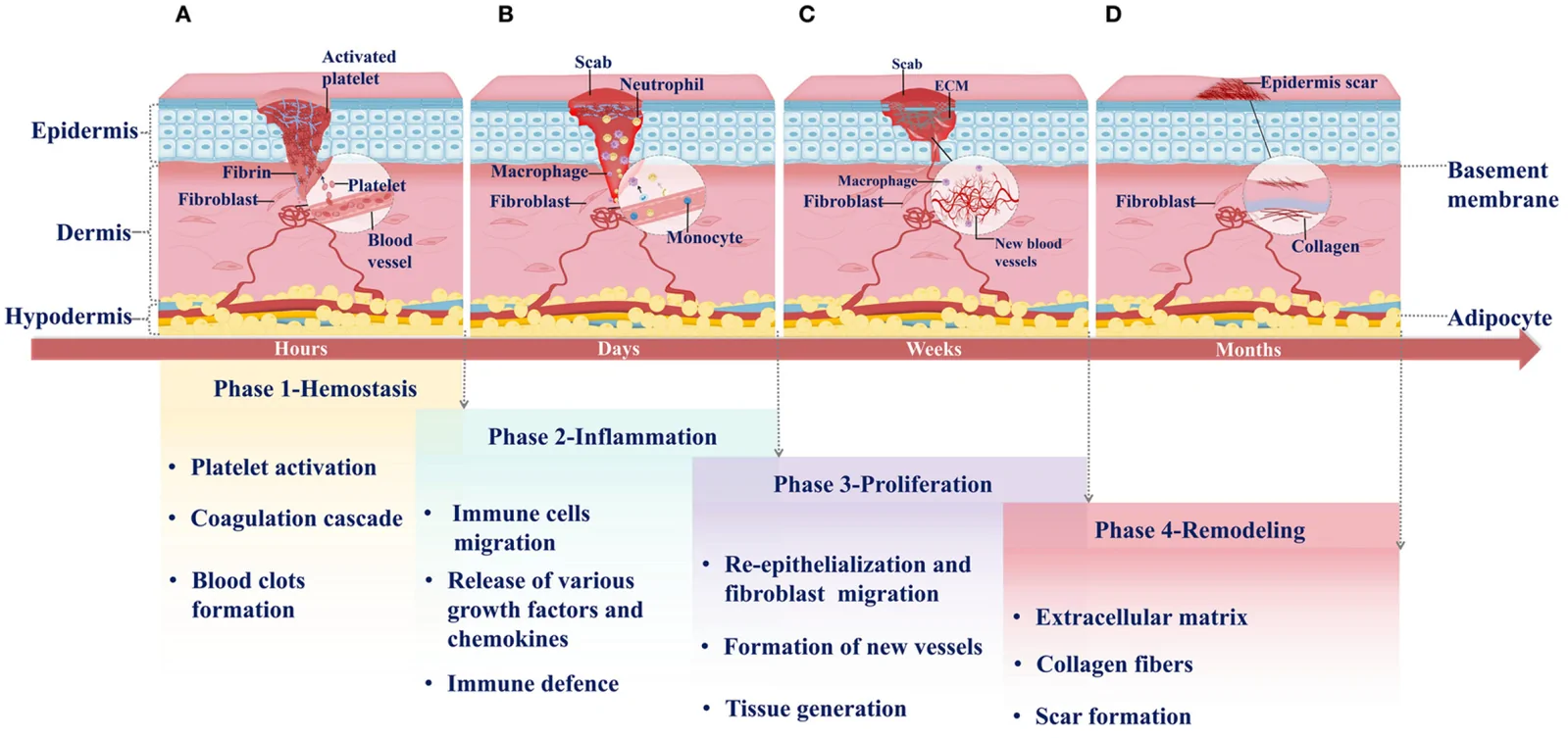
Dynamic evolution of macrophages and core cellular events across the four overlapping phases of cutaneous wound healing. (**A**) Hemostasis phase: platelets aggregate to form blood clots immediately after injury, and polymorphonuclear neutrophils are recruited from peripheral vessels as the first-line immune cells. (**B**) Inflammatory phase: circulating CCR2^+^Ly6C^+^ monocytes infiltrate the wound bed and differentiate into classically activated M1 macrophages, which eliminate pathogens, PAMPs and DAMPs through production of nitric oxide (NO), reactive oxygen species (ROS) and pro-inflammatory cytokines including TNF-α, IL-1α, IL-1β, IL-6, IL-12, CXCL9 and CXCL10. (**C**) Proliferation phase: macrophages undergo functional repolarization toward a pro-reparative M2-like phenotype; they secrete tissue-repairing mediators (TGF-β, IL-10, IL-1ra, PDGF, VEGF) to drive fibroblast proliferation, neovascularization and re-epithelialization. (**D**) Remodeling phase: macrophage-derived matrix metalloproteinases (MMPs) orchestrate ECM remodeling and collagen fiber maturation to form stable scar tissue. Reproduced from Ref. [23]. Licensed under CC BY 4.0.

All stages of wound healing are regulated by macrophages, which exert vital biological functions throughout the repair process. Early-stage pro-inflammatory macrophages (M1-like) serve two principal functions: they secrete pro-inflammatory cytokines such as TNF-α and IL-1β, and clear apoptotic neutrophils via efferocytosis. Once inflammatory activity subsides, macrophages gradually acquire pro-reparative (M2-like) functions. The VEGF and PDGF they produce encourage blood vessel formation and tissue recovery, which lays the groundwork for the proliferative phase of wound repair [6,22,23] (Figure 2).

Granulation tissue formation, neovascular growth and epithelial regrowth represent the three defining characteristics of the proliferative wound repair phase. Once triggered, fibroblasts start generating type III collagen and fibronectin, which together assemble granulation tissue. Guided by VEGF molecular signals, endothelial cells grow new branches and build interconnected capillary networks. Meanwhile, keratinocytes move outward from wound margins, multiply rapidly, and ultimately re-cover the exposed wound surface [24,25]. Collagen fibers are reorganized and cross-linked under physical tension, which eventually creates fully formed scar tissue. At the same time, the weaker type III collagen is gradually replaced by tougher type I collagen. All these structural adjustments happen in the final remodeling stage, a process that can extend over months or even years [26].

### 2.2. Coregulatory Network of M1/M2 Macrophage Polarization

The functional states of macrophages can be continuously reshaped by diverse microenvironmental stimuli. The M1 inflammatory subtype and M2 tissue-repair subtype are regarded as the two extreme states along this continuous polarization gradient. Interferon-γ (IFN-γ), lipopolysaccharide (LPS) and TNF-α act as key stimuli that trigger the formation of M1 macrophages, which carry surface biomarkers CD86 and inducible nitric oxide synthase (iNOS). This macrophage subtype is characterized by three functional traits: enhanced antigen presentation, complement-assisted phagocytosis, and robust release of NO and pro-inflammatory cytokines including TNF-α, IL-1β, IL-6 and IL-12.

IL-4, IL-13 and IL-10 promote the differentiation of monocytes toward an M2-like phenotype, characterized by CD206 and Arg-1 expression [27]. These cells predominantly produce anti-inflammatory molecules such as IL-10 and TGF-β, which facilitate tissue repair, promote angiogenesis, and support ECM remodeling [28].

While the M1/M2 nomenclature provides a useful heuristic framework, it is increasingly recognized that macrophage polarization in vivo rarely conforms to the rigid dichotomy observed under idealized in vitro conditions. Instead, macrophages frequently adopt transitional or mixed phenotypes that co-express both pro-inflammatory and anti-inflammatory markers, particularly in the complex microenvironment of chronic wounds. Disease-specific macrophage states have also been documented: for instance, macrophages in diabetic wounds exhibit a unique metabolic–inflammatory phenotype that does not fully align with either classical M1 or M2 classifications. Therefore, the M1/M2 terminology used throughout this review should be interpreted as a continuum of functional states rather than discrete cellular subsets, and references to ‘M1-like’ and ‘M2-like’ phenotypes are intended to describe directional shifts in functional polarity, not fixed cell populations.

A stable ratio of M1 versus M2 macrophage subtypes relies on precise control from a network of mutually connected signaling pathways. Major inflammatory signaling pathways such as NF-κB and MAPK drive macrophages toward an M1-like phenotype [29]. By contrast, other signaling pathways guide cells to become repair-focused M2 macrophages. The best-studied ones are STAT6, PPARγ and the PI3K/Akt signaling system [30,31,32].

M2 macrophages are functionally heterogeneous. Based on what activates them and what roles they play, researchers split M2 macrophages into three subtypes, known as M2a, M2b and M2c [28]. IL-4 and IL-13 trigger the M2a subtype, which helps repair damaged tissue. Immune complexes turn on M2b macrophages to regulate local immune responses. As for M2c cells, they form when IL-10 and TGF-β act together, working to reduce inflammation and rebuild tissue structure. For narrative consistency, this review uniformly uses “M2 phenotype” to describe the anti-inflammatory/pro-reparative polarization state induced by polysaccharides. In addition, metabolic reprogramming has emerged as an independent regulatory dimension beyond classical signaling pathways. Subsequent sections distinguish between two mechanistic modes of polysaccharide-induced M2 polarization: classical signaling pathway regulation and metabolic reprogramming modulation [33,34], to clarify the depth of current mechanistic research.

Beyond classical signaling cascades and metabolic reprogramming, recent advances in single-cell and spatial transcriptomics have substantially refined our understanding of macrophage heterogeneity and the spatiotemporal dynamics of the wound immune microenvironment [35]. Zandigohar et al. integrated single-cell RNA sequencing with single-cell ATAC sequencing to infer transcription factor (TF) activity in wound monocytes/macrophages during murine skin repair. Their analysis revealed that wound macrophages clustered into three distinct populations—early-stage monocytes/macrophages, late-stage macrophages, and antigen-presenting cells—each exhibiting differential chromatin accessibility and predicted TF activity that did not always correlate with mRNA or protein expression [36]. Network analysis identified two highly interconnected TF communities involving 19 transcription factors, with *Nr4a1* and *Nfkb1* co-populating a pro-inflammatory subnetwork. Functional validation demonstrated that *Nr4a1* knockout mice exhibited decreased inflammatory gene expression in early-stage wound macrophages, alongside delayed re-epithelialization and impaired granulation tissue formation, underscoring the functional relevance of TF networks in dictating macrophage phenotype and healing outcomes [37].

In the human context, Liu et al. constructed a spatiotemporal single-cell roadmap of acute skin wound healing by performing multi-omics analyses on serial biopsies from the same individuals across inflammation, proliferation, and remodeling phases. This landmark study revealed that pro-inflammatory macrophages and fibroblasts sequentially support keratinocyte migration in a relay-like manner across different healing stages. When comparing this acute wound atlas with single-cell data from venous and diabetic foot ulcers, the authors identified a link between failed keratinocyte migration and impaired inflammatory responses in chronic wounds [38]. Intriguingly, spatial transcriptomics in diabetic foot ulcer patients revealed a higher abundance of M1 macrophages in healing wounds, whereas M2 macrophages predominated in non-healing ulcers—a finding that challenges the simplistic assumption that M2 polarization is universally beneficial and highlights the importance of contextualizing macrophage phenotypes within the precise spatial and temporal architecture of the wound bed. Collectively, these single-cell and spatially resolved approaches are moving the field beyond reductionist M1/M2 classifications, revealing that macrophage functional states exist along a continuum shaped by both intrinsic transcriptional programs and extrinsic niche signals [36,39]. For the design of polysaccharide-based immunomodulatory hydrogels, these insights underscore the need to consider not only the polarity of macrophage polarization but also the precise timing, spatial distribution, and cellular crosstalk that govern effective tissue repair.

### 2.3. M1/M2 Imbalance in Chronic Wounds: A Key Target for Treatment

For hard-to-heal wounds such as diabetic foot ulcers, venous leg ulcers and pressure sores, macrophages fail to maintain the proper balance between pro-inflammatory and pro-reparative functional states. This imbalance is characterized by persistent predominance of the M1-like phenotype and impaired M2-like functional capacity, leading to a prolonged inflammatory phase that fails to transition into the proliferative phase [40].

In diabetic ulcer tissue, pro-inflammatory cascades such as NF-κB get triggered by persistent AGE buildup and high blood glucose levels [34]. Meanwhile, pathways responsible for M2 macrophage differentiation (STAT6 as a typical example) receive consistent suppression [33]. This results in an abnormal increase in M1-like macrophages and impaired M2-like macrophage recruitment and function [41]. Beyond phenotypic imbalance, M2 macrophages in chronic wounds also exhibit defective efferocytosis—the ability to clear apoptotic neutrophils and necrotic tissue debris—a defect that further amplifies local inflammation and forms a vicious cycle. Inflammation that continuously damages tissue creates a self-reinforcing inflammatory cycle [42,43]. Under this condition, wounds fail to heal as they should, eventually turning into chronic ulcers that fail to close completely.

Emerging evidence highlights the role of metabolic dysfunction in perpetuating M1/M2 imbalance. Macrophages inside diabetic wounds show abnormal metabolic activity: glycolysis stays continuously active while mitochondrial oxidative phosphorylation becomes dysfunctional. These metabolic defects trap macrophages within an inflammatory phenotype [44]. Sustained overactivation of the NF-κB signaling axis is driven by growing concentrations of TNF-α and IL-1β. This chain of molecular events establishes a self-reinforcing inflammatory cycle that cannot be easily terminated.

Macrophages control every step of skin wound healing. Therefore, therapies that reprogram macrophages from a pro-inflammatory (M1-like) toward a pro-reparative (M2-like) phenotype and adjust the local immune state to help tissue regrow have become the main treatment for non-healing chronic ulcers [45]. Notably, exogenous immunomodulatory agents—including specific natural polysaccharides—have shown promise in reprogramming macrophage polarization by targeting the receptors and signaling pathways described above, forming the mechanistic basis for the advanced wound dressings discussed in the following sections [13,46].

## 3. Immunomodulatory Natural Polysaccharides and Their Wound Dressing Constructs

Owing to their excellent biocompatibility, degradable properties and inherent capacity to adjust immune reactions, natural polysaccharides are widely regarded as outstanding raw materials to fabricate wound dressings with multiple biological functions. Polysaccharides form the main frame of wound dressings and offer mechanical support for tissue regrowth. They can also attach to recognition proteins on macrophage surfaces and act as bioactive regulators. This interaction balances immune conditions in wounds and guides macrophages to turn into repair-focused M2 cells. We can divide natural polysaccharides that push macrophages toward the repair-friendly M2 type into two major groups by their molecular makeup, focusing on monosaccharide components and the way sugar units link together. One group contains mannose or mannose-like segments, and the other does not. These polysaccharides can be formulated into various wound care products, including hydrogels, sponges, fibrous sheets, and microneedles, which have shown efficacy in acute wounds, chronic ulcers, and tissue regeneration models.

### 3.1. Polysaccharides Categorized as Mannose-Containing Based on Operational Criteria

Polysaccharides containing mannose or mannose-like units possess unique advantages in modulating macrophage phenotypes, primarily by serving as ligands for the mannose receptor (MR, CD206) or other mannose-recognizing C-type lectin receptors such as SIGNR1. It is important to note that the inclusion of a given polysaccharide in this category is operationally determined by a dual criterion: (i) a mannose content ≥ 20% of total monosaccharides, and (ii) immunomodulatory activity mediated specifically through mannose-recognizing receptors (e.g., MR). This definition explicitly excludes C-type lectins like Dectin-1, which recognize β-glucan backbones rather than mannose motifs, even though they belong to the same receptor superfamily.

For each polysaccharide discussed below, receptor engagement evidence is classified as direct (supported by binding assays, receptor knockdown, or blocking experiments) or indirect (inferred from phenotypic correlation or pathway inhibition). The latter does not establish definitive causation.

Strict application of this threshold is, however, subject to mechanistic nuance. For borderline cases in which mannose content approaches but does not reach the 20% cutoff, inclusion may be justified only if mannose residues are positioned on solvent-exposed side chains with demonstrated spatial accessibility to MR, and supported by direct receptor-binding evidence. Such exceptions are explicitly flagged as “mechanistic borderline cases” to prevent categorical misclassification. This nuanced approach ensures that the classification remains structurally grounded while accommodating biologically relevant exceptions.

Distinct from typical high-mannose glucomannans and the mechanistic borderline case GLP, LBP merely contains trace mannose (5–10 mol%) without MR-dependent immunoregulation. Its capacity to promote M2 polarization originates from complex arabinogalactan branches that negatively regulate NF-κB/MAPK inflammatory pathways, rather than mannose-mediated receptor recognition.

#### 3.1.1. Konjac Glucomannan

As a water-soluble neutral acetylated glucomannan, KGM is isolated from konjac tuber tissues. β-1,4 glycosidic bonds link D-glucose and D-mannose to construct KGM’s main molecular chain. Minor structural branches are introduced by two bonding modes: β-1,3 bonds on the C-3 position of mannose residues, and β-1,6 linkages that anchor extra glucosyl side segments. Differences in konjac strains and extraction protocols alter the mannose/glucose molar ratio, which fluctuates between 1.5:1 and 2:1 and hits an average value of 1.6:1 in most samples. Random substitution of acetyl groups takes place on the C-6 carbon of each sugar residue, with a substitution frequency of one acetyl group per 9 to 19 sugar units. These acetyl modifications substantially influence KGM’s solubility, chain conformation, and gelation properties [47,48].

Konjac glucomannan oligosaccharides (KMOS), the breakdown fragments of KGM, rely on SIGNR1 signal transduction to suppress inflammatory reactions within intestinal tissue (mechanistic corroboration for conserved receptor signaling only). The signaling cascade consisting of phospho-c-Raf(S338), phospho-p65(S276) and acetyl-p65(K310) gets triggered by KMOS, which remodels macrophage phenotypes and facilitates an M2-like phenotypic shift in pro-inflammatory macrophages [49].

KGM has been extensively investigated as a hydrogel wound dressing material, leveraging its capacity to modulate local immune activity. A typical composite material mixes KGM with Bletilla striata polysaccharide (BSP) and uses 1,4-butanediol diglycidyl ether (BDDE), as the crosslinking agent. Compared with KGM-only hydrogels, this blended material exhibits greater water uptake, enhanced mechanical strength (maximum tensile stress: 6.6 kPa), and superior tissue adhesion. In vitro studies demonstrate this composite hydrogel slows down the TNF-α/NF-κB inflammatory pathway and boosts the production of IL-10, a molecule that reduces inflammation. Together, these two effects ease inflammatory damage and help damaged skin tissue regrow faster [50]. In this composite system, both KGM and BSP function synergistically as immunomodulatory agents, while the crosslinked network serves primarily as a structural scaffold.

Furthermore, researchers developed a straightforward two-step fabrication process to produce porous sponge composites mixing KGM and silver nanoparticles (KGM/AgNP). This porous dressing material absorbs and locks in large volumes of water, exhibits favorable mechanical properties, and can strongly suppress bacterial growth. When tested on lab animals, the composite speeds up wound recovery by stimulating fibroblast proliferation and driving full epithelial layer reconstruction [51]. Here, the KGM matrix provides a porous structural scaffold and contributes to immunomodulation, whereas AgNPs act as the antibacterial component.

Additionally, in trials using full-thickness skin wounds on mice, acetylated konjac glucomannan (AceKGM) fiber membranes greatly shorten wound recovery time by stimulating epithelial regrowth, tissue reconstruction and collagen generation [52]. This beneficial healing effect comes from the material’s ability to adjust macrophage function: macrophages cultivated on the AceKGM fiber film release more cytokines that suppress inflammation and facilitate tissue regeneration. This mechanistic insight provides the rationale for employing AceKGM fibrous membranes as immunomodulatory wound dressings.

#### 3.1.2. *Bletilla striata* Polysaccharide

*Bletilla striata* is a medicinal plant widely used in traditional Chinese medicine. Its main active substance is a water-soluble neutral glucomannan, hereafter referred to as BSP. It consists of D-mannose and D-glucose with a molar ratio of approximately 3:1. D-mannopyranose and D-glucopyranose residues are linked via β-1,4 bonds to construct the polymer’s primary skeleton. Minor short side chains are bonded to the C-3 position of a portion of mannose structural units [53]. This linear and low-branched glucomannan possesses favorable film-forming, hemostatic and anti-inflammatory capacities, and serves as an excellent skeleton material for wound-healing hydrogels.

In mouse models of diabetic ulcers, a borate-crosslinked hydrogel made from BSP, berberine and borax achieved 94% wound closure in damaged skin tissue [54]. Two core biological functions support this healing outcome: first, it eliminates more than 90% of *Escherichia coli* and *Staphylococcus aureus* pathogens; second, it reduces NO synthesis, thereby mitigating inflammatory damage. In this system, BSP serves as the primary immunomodulatory entity and structural scaffold, while berberine contributes antibacterial activity.

Apart from this chemically crosslinked self-healing system, a simple physical blend of BSP, carboxymethyl chitosan (CMCS) and Carbomer 940 is efficacious in wound management [55]. This mixed hydrogel forms porous internal structures that grant elasticity and stable water retention, which enables efficient recovery of full-thickness skin lesions. Here, BSP and CMCS co-function as both structural scaffold and immunomodulatory components.

#### 3.1.3. *Ganoderma lucidum* Polysaccharide

GLP is a highly branched heteropolysaccharide and the major bioactive constituent of *Ganoderma lucidum*. Its monosaccharide composition is dominated by glucose (60–70%), followed by mannose (10–20%), along with smaller amounts of galactose and arabinose. The central polymer framework consists of a β-1,3-linked D-glucan backbone, with glucose or mannose residues attached as side segments via β-1,6 glycosidic connections. The branching degree and monosaccharide ratio vary with fungal strains and extraction methods [56]. This distinctive architecture enables GLP to engage multiple pattern recognition receptors on macrophages: the β-1,3-glucan backbone is recognized by Dectin-1, while the β-1,6-linked mannose side chains are accessible to the MR, thereby supporting dual-receptor engagement.

A double-network wound dressing was made from oxidized GLP, carboxymethyl cellulose (CMC) and sodium alginate (SA). When this material is applied, it reduces cellular reactive oxygen species (ROS) levels and attenuates pro-inflammatory markers while upregulating M2-associated anti-inflammatory markers in stimulated macrophages. The combination of these two benefits reduces inflammation and significantly accelerates the healing of diabetic skin wounds [57]. In this double-network system, GLP acts as the immunomodulatory agent, whereas SA and CMC provide the structural scaffold.

**Figure 3:**
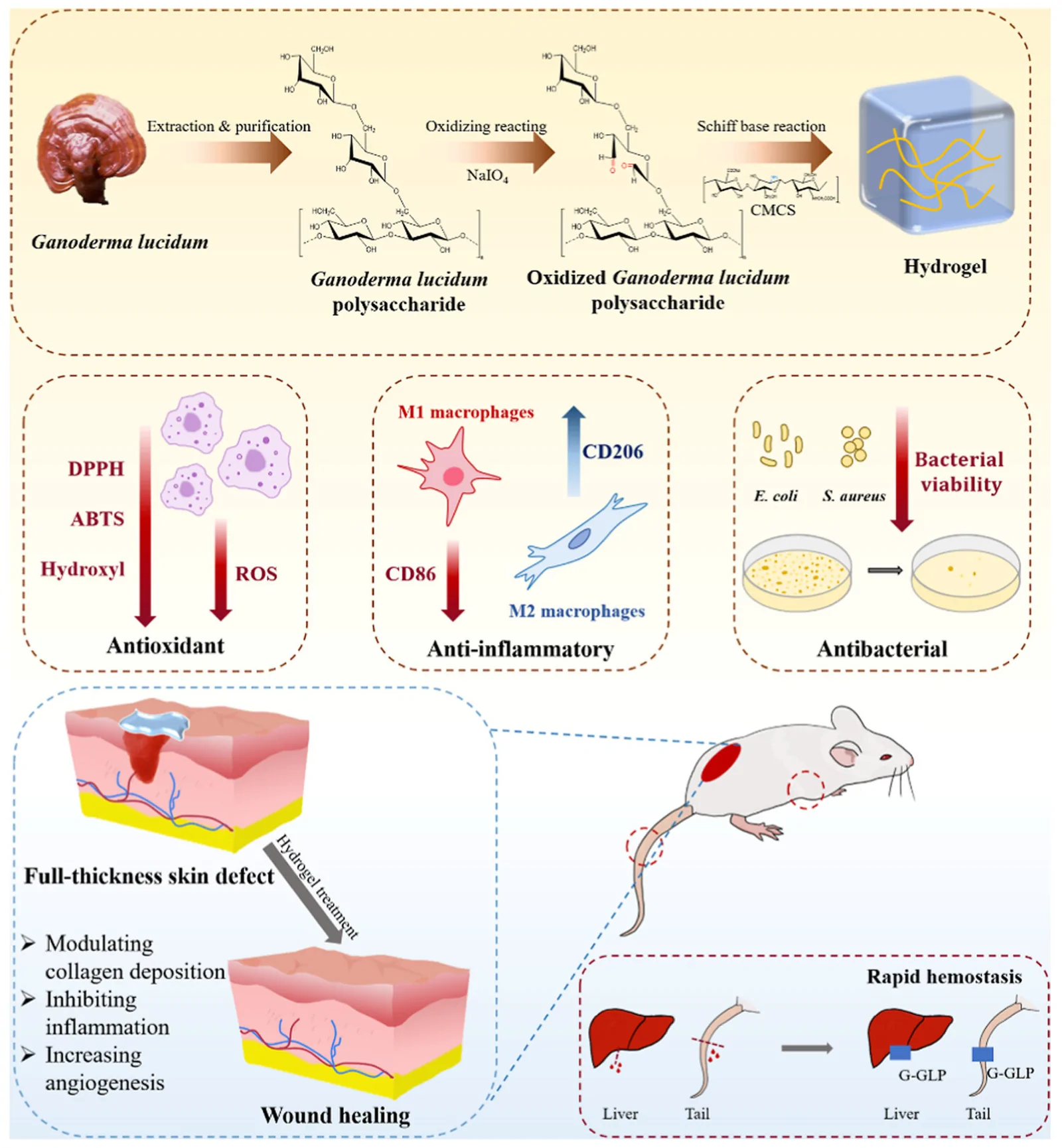
Schematic illustration of G-GLP hydrogel fabrication and its integrated therapeutic functions for skin wound repair. The hydrogel network is formed via Schiff base reaction between oxidized Ganoderma lucidum polysaccharide (OGLP) and CMCS, without additional chemical crosslinkers. It exerts synergistic antioxidant, anti-inflammatory and antibacterial activities, drives macrophage polarization toward the repair-type M2 phenotype, and ultimately promotes full-thickness skin tissue regeneration, with additional hemostatic capacity. Reproduced with permission from Ref. [58]. Copyright 2025, American Chemical Society.

Zou et al. further developed a series of crosslinker-free GLP-based hydrogels (G-GLP) through Schiff base crosslinking between oxidized GLP and CMCS [58]. The optimized hydrogel exhibited tunable mechanical properties, favorable swelling-degradation behavior and good cytocompatibility. It effectively scavenged intracellular ROS, downregulated the M1 biomarker CD86 and upregulated the M2 marker CD206 in LPS-stimulated macrophages. In vivo studies verified that the hydrogel accelerated full-thickness skin wound healing via inflammation alleviation, angiogenesis promotion and collagen deposition, and also showed superior hemostatic performance in acute bleeding models (Figure 3).

Subsequently, cystine dihydrochloride serves as a crosslinker to bind oxidized Ganoderma lucidum polysaccharide (OGLP) and ferulic acid, generating carrier-free Ganoderma lucidum polysaccharide–ferulic acid nanoparticles (GFNPs). A thermosensitive chitosan–poloxamer polymer (CPP) hydrogel scaffold consisting of CS and poloxamer can encapsulate both GFNPs and recombinant human epidermal growth factor (rhEGF) simultaneously. Powerful ROS clearance, M2 macrophage induction and broad antibacterial capacity are all provided by GFNPs. Combined with rhEGF, the composite system produces synergistic therapeutic outcomes that stimulate angiogenesis and accelerate tissue restoration in diabetic ulcer animal models [59]. In this system, GLP-derived GFNPs function as the immunomodulatory and antioxidant entity, rhEGF serves as the pro-angiogenic growth factor, and the CPP hydrogel acts as a thermoresponsive delivery vehicle.

TiO_2_–Ag nanoparticles and GLP biomacromolecules are embedded within the outer shell, and iron oxide nanoparticles are sealed inside the inner core of polycaprolactone yolk–shell structures (YSPs). This type of multi-purpose particulate material is manufactured via trineedle coaxial electrospray technology. These YSPs demonstrated antibacterial, antioxidant, and photothermal effects, showing promising wound-healing effects in a burn wound model [60]. Here, GLP embedded in the outer shell contributes immunomodulatory activity, while the yolk–shell architecture functions as a multi-compartment delivery vehicle for synergistic therapy.

#### 3.1.4. *Dendrobium officinale* Polysaccharide

*Dendrobium officinale* polysaccharide (DOP) is a typical highly acetylated glucomannan and the core bioactive substance of Dendrobium officinale. The molar ratio of mannose to glucose in DOPS ranges from 3:1 to 5:1, with mannose being the dominant monosaccharide. D-mannopyranose and D-glucopyranose units link together through β-1,4 glycosidic bonds to form the main chain of this polysaccharide. Acetyl groups are mostly attached to the C-2 and C-3 positions of mannose units on the chain. The degree of acetylation substantially influences the biological activity of the polysaccharide [61].

In a non-cutaneous colitis model (mechanistic corroboration for metabolic reprogramming only), activation of the SENP1-SIRT3 signaling cascade triggered by DOPS restrains glycolytic metabolism inside macrophages. This metabolic suppression promotes a phenotypic shift toward an anti-inflammatory, M2-like state in macrophages under inflammatory conditions, which ultimately relieves inflammatory responses [62].

**Figure 4:**
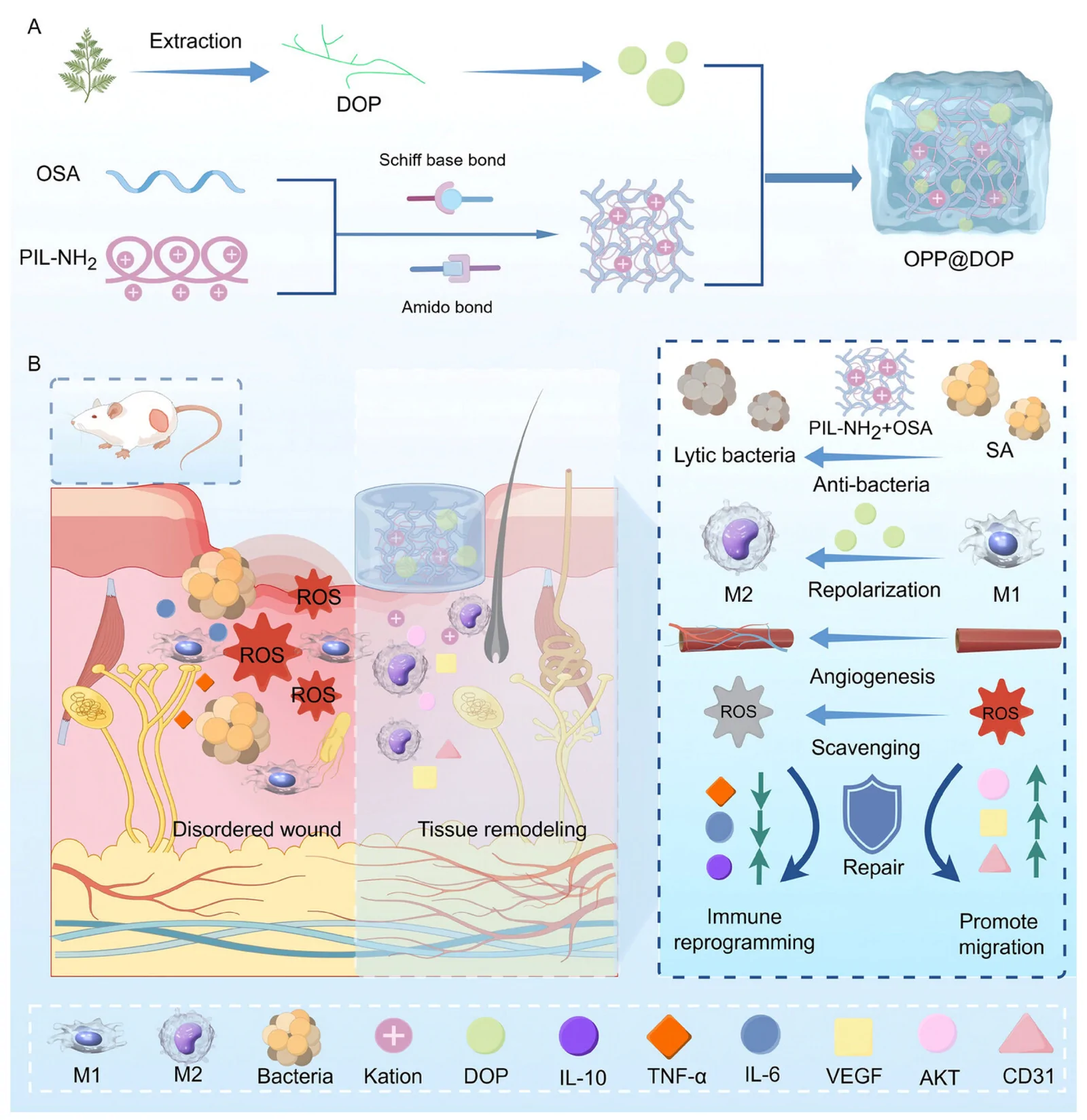
Schematic diagram of the all-in-one DOP-loaded ionic hydrogel (OPP@DOP) for diabetic wound healing. (**A**) This panel illustrates how OPP@DOP is fabricated: oxidized sodium alginate (OSA) reacts with amino-modified imidazole salts (PIL-NH_2_) to create dynamic Schiff base and amide crosslinks, while DOP molecules are incorporated into the crosslinked network. (**B**) Four coordinated therapeutic effects support wound recovery after hydrogel administration: cationic interactions eliminate MRSA bacteria, surplus ROS get neutralized, macrophages shift toward the anti-inflammatory M2 subtype, and the AKT signal cascade is activated to stimulate new blood vessel growth. Reproduced with authorization from Ref. [63]. Copyright 2025, Wiley-VCH Verlag GmbH & Co. KGaA, Weinheim.

Dynamic Schiff base and amide linkages connect oxidized sodium alginate (OSA) and imidazole salt molecules to fabricate an all-in-one ionic hydrogel system. DOP molecules are fixed within this network relying on electrostatic forces, yielding the final OPP@DOP hydrogel. The material exhibits multiple desirable properties: favorable biocompatibility, strong water retention capacity, favorable tissue adhesion, antioxidant capacity, electrical conductivity, and remarkable bacteriostatic effects toward methicillin-resistant Staphylococcus aureus (MRSA). Sustained DOP release exerts multiple beneficial biological effects. It reduces inflammation and clears excess ROS, activates the AKT pathway to promote blood vessel formation, and favors macrophage reprogramming from a pro-inflammatory toward a pro-resolving phenotype. In this all-in-one system, DOP acts as the principal immunomodulatory agent, while the OSA-PIL-NH_2_ network provides both structural scaffold and ionic conductive properties. Collectively, these effects accelerate skin wound healing [63] (Figure 4).

#### 3.1.5. *Aloe vera* Polysaccharide

The principal bioactive component of *Aloe vera* polysaccharide is acemannan. D-mannopyranose residues are linked through β-1,4 bonds to construct the linear molecular backbone of acetylated mannan known as acemannan. Acetyl functional groups are bound to the C-6 site of one mannose unit in every 2–3 sugar residues. Minor side-chain segments containing galactose and glucuronic acid are grafted onto the primary chain, and mannose constitutes more than 70% of the total monosaccharide content [64]. Acemannan exhibits anti-diabetic activity through inhibition of inflammatory cytokines and apoptotic pathways [65].

Aloe polysaccharide and collagen are mixed to produce a novel porous composite wound dressing abbreviated AP-CD. Suppression of inflammatory activity, accelerated angiogenesis, elevated growth and anti-inflammatory factor expression, and reduced pro-inflammatory cytokine concentrations can all be observed in vivo after AP-CD administration. A higher percentage of macrophage cells can be detected within AP-CD-treated wound tissue through single-cell sequencing [66]. Here, aloe polysaccharide primarily functions as an immunomodulatory agent driving M2 polarization, whereas collagen provides the structural scaffold for tissue ingrowth.

Dual Schiff-base crosslinking between phenylboronic acid (PBA)-modified HA and oxidized aloe polysaccharide (OAP) is adopted to fabricate a stimuli-responsive wound hydrogel carrying phycocyanin-functionalized FeS nanoparticles, named HA-PBA/OAP/FeS@PC. Injectability and self-repair capacity are intrinsic features of this material, alongside its capacity to sense pH, glucose and ROS fluctuations. Under 660 nm laser illumination, photothermal conversion, Fenton-derived hydroxyl radical production and hydrogen sulfide release are activated in tandem. These combined actions eliminate bacteria and mitigate inflammatory reactions within animal models of infected diabetic wounds [67]. In this multi-component hydrogel, HA and OAP serve dual roles as both structural scaffold and immunomodulatory entities, while FeS@PC nanoparticles function as photothermal and chemodynamic therapeutic agents.

Additionally, electrospun nanofiber sheets fabricated from aloe polysaccharides blended with polyvinyl alcohol (PVA) and SA, incorporating green-synthesized AgNPs, displayed a uniform structure, sustained silver ion release, strong antibacterial activity, and promoted angiogenesis and wound healing in a burn wound model [68].

#### 3.1.6. Polysaccharide X21 from *Anoectochilus xingrenensis*

Polysaccharide X21 is a uniform polysaccharide isolated from *Anoectochilus xingrenensis*. Its long molecular backbone is composed of three glycosidic units: →4)-β-D-Galp-(1→, →4)-α-D-Glcp-(1→ and →4,6)-α-D-Glcp-(1→, while α-D-Manp caps the ends of side branches. The molar proportion of galactose, glucose and mannose within this polymer is roughly 2.5 to 1.8 [69].

In a non-cutaneous arthritis model (mechanistic corroboration only), when tested on rats with collagen-triggered arthritis, X21 reshaped the balance of intestinal flora: it lowered the proportion of Lactobacillus bacteria and raised the population of Roseburia, which further elevated the production of short-chain fatty acids.

Both in vitro and in vivo studies demonstrated that X21 inhibits M1 polarization and promotes M2-like anti-inflammatory polarization. This regulatory effect may rely on two sets of molecular changes: reducing the expression of phosphorylated p65, IκB, JNK and p38 to inhibit NF-κB and MAPK signaling, and boosting phosphorylated STAT6 to activate the JAK/STAT pathway [69].

#### 3.1.7. *Periplaneta americana* Polysaccharide

A homogeneous polysaccharide, designated PAP55-3-1, was isolated from *Periplaneta americana*. Its monosaccharide components include glucose, galactose, arabinose and mannose (6.5 mol%), with a molar ratio of approximately 5.2:2.8:1.35:0.65. Two types of glycosidic bonds make up the polysaccharide’s core framework: α-1,4 bridges for glucose residues, and β-1,3 linkages for galactose residues. PAP55-3-1 shields mitochondria from oxidative injury triggered by hydrogen peroxide. In RAW264.7 macrophage cells, this polysaccharide shifts the immune balance toward a pro-resolving, M2-like state by restraining M1-type differentiation, thereby delivering dual benefits of relieving inflammation and accelerating tissue regeneration [70]. Follow-up mechanistic research uncovered that PAP55-3-1 activates the hexosamine biosynthesis pathway (HBP). This signaling cascade boosts site-specific O-GlcNAc modification of HIF-1α protein, which then elevates PPARγ expression and sustains steady M2 macrophage polarization [71].

CS thin films loaded with purified PAP55-3-1 were tested on mouse cutaneous wound models. Marked improvements in skin lesion repair were observed after applying this biomaterial. CS and polysaccharide residues separated from Periplaneta americana are co-formulated to create the Periplaneta americana remnant polysaccharide-chitosan composite film (PAPCF). Favorable biocompatibility, bacteriostatic performance and antioxidant capacity can all be verified for PAPCF. It accelerates wound contraction and collagen buildup, thereby facilitating the transition of skin lesions from the inflammatory phase to the proliferative phase of repair [72]. In this composite film, CS acts primarily as a structural scaffold with adjunctive antibacterial effects, whereas Periplaneta americana remnant polysaccharide (PAP) serves as the principal immunomodulatory component driving the anti-inflammatory response.

#### 3.1.8. Polysaccharide from *Schizophyllum commune*

The F2 fraction of polysaccharide extracted from *Schizophyllum commune* is a hyperbranched mannan, containing 55.2 mol% mannose, 28.7 mol% glucose and a small amount of galactose. Glucose and galactose form the terminal segments of every side branch of this polysaccharide. Branching structures originate from (1→2,3)-linked mannopyranose units, and the long central chain consists of mannopyranose residues connected by (1→3) bonds. Secretion of NO and assorted cytokines from RAW264.7 macrophages is driven by simultaneous activation of NF-κB and MAPK pathways. This whole signaling cascade starts when the polysaccharide engages CR-3 and TLR-4 receptors to trigger cellular activation [73,74].

#### 3.1.9. Concluding Remarks of Mannose-Containing Polysaccharides

Collectively, mannose-containing polysaccharides exert anti-inflammatory/M2-polarizing activity primarily by targeting C-type lectin receptors (MR, Dectin-1, SIGNR1) on macrophage surfaces, yet their downstream signaling pathways and functional potency differ significantly. For linear glucomannans (KGM, BSP, DOPS), the density of mannose residues on the backbone and the degree of acetylation are key structural determinants of immunomodulatory activity: higher mannose ratio and moderate acetylation have been proposed to enhance receptor binding affinity, although systematic structure–activity studies with purified, structurally defined fractions are still lacking. For branched heteropolysaccharides (GLP, LBP, X21), their complex spatial conformation and multi-site recognition enable simultaneous modulation of multiple pathways (e.g., NF-κB inhibition and STAT6 activation), achieving more comprehensive immune regulation.

It should be noted that extraction-derived contaminants (e.g., endotoxin, residual proteins, polyphenols) and batch-to-batch variations in molecular weight and branching can profoundly confound macrophage responses, making definitive structure–activity conclusions premature. Beyond inherent structural variations, extrinsic factors such as extraction procedures and residual impurities also confound immunomodulatory outcomes. Differences in extraction temperature, solvent, and purification steps alter molecular weight distribution and branching architecture, affecting receptor accessibility and signaling potency [75]. Meanwhile, trace endotoxin contamination can artificially amplify pro-inflammatory responses via TLR4, leading to overestimation of a polysaccharide’s intrinsic activity. These variables are rarely systematically controlled across studies, hindering reliable structure–activity comparisons [76].

From the perspective of action mode, most mannose-containing polysaccharides directly regulate macrophage phenotype through receptor–ligand recognition, while a few achieve indirect immunomodulation by scavenging ROS or exerting antibacterial effects. Metabolic remodeling to control M2 macrophage polarization has only been documented for a limited set of polysaccharides including DOPS and Periplaneta americana-derived polysaccharide. For most other research work, analyses are restricted to the observational description of canonical signaling pathways. Notably, most mannose-rich polysaccharides exhibit moderate gel-forming properties and are often used as functional active components in composite hydrogel systems rather than sole scaffold materials.

### 3.2. Polysaccharides Without Mannose Structural Units

#### 3.2.1. Chitosan

CS is the only naturally occurring polysaccharide that possesses cationic properties, derived from the deacetylation of chitin. Its main chain consists of randomly arranged β-1,4-linked D-glucosamine and N-acetyl-D-glucosamine residues. The degree of deacetylation (DDA), generally 70–95% for commercial CS, is the core structural parameter that determines the positive charge density, water solubility and biological activities such as antibacterial and hemostatic effects [77]. The cationic property is the fundamental basis for its functions: CS adsorbs negatively charged bacteria, erythrocytes and platelets through electrostatic interaction to exert antibacterial and hemostatic effects, and interacts with macrophage receptors to regulate inflammatory pathways and promote an anti-inflammatory macrophage profile. These effects have been characterized primarily in LPS-stimulated macrophages, indicating a phenotypic rebalancing rather than a unidirectional switch.

##### Self-Healing and Injectable Hydrogels

Catechol-functionalized chitosan (CFCS) and bioactive vanillin serve as raw materials for fabricating the PAAc/CFCS-Vanillin hydrogel wound dressing. This gel exhibits satisfactory mechanical robustness and superior adhesive capacity, together with reliable antibacterial performance. Excess ROS clearance and inflammatory factor modulation are achieved by vanillin, promoting an anti-inflammatory macrophage phenotype; such integrated functions support its wide application prospect for chronic diabetic wound therapy [78].

Guanidinium-functionalized chitosan (GCS) and aldehyde-modified chitosan (ACS) were synthesized, and self-healing chitosan hydrogels (SCgels) were prepared via the Schiff base reaction. This hydrogel quickly eliminated bacteria in infected wounds and reduced inflammation, achieving a 94.5% wound closure rate in infected full-thickness rat wounds [79].

Quaternized chitosan acts as the base material to form a self-assembled supramolecular hydrogel loaded with hesperidin, which is named CQHP. This material is fabricated to act as a photothermal antibacterial patch to treat MRSA-infected skin lesions. CQHP hydrogel exhibits injectability, self-healing capability, favorable adhesion, antioxidant activity, hemostatic properties, and protein adsorption capacity. These integrated properties remarkably speed up the closure of MRSA-contaminated wounds.

##### Multifunctional and Biomimetic Hydrogels

**Figure 5:**
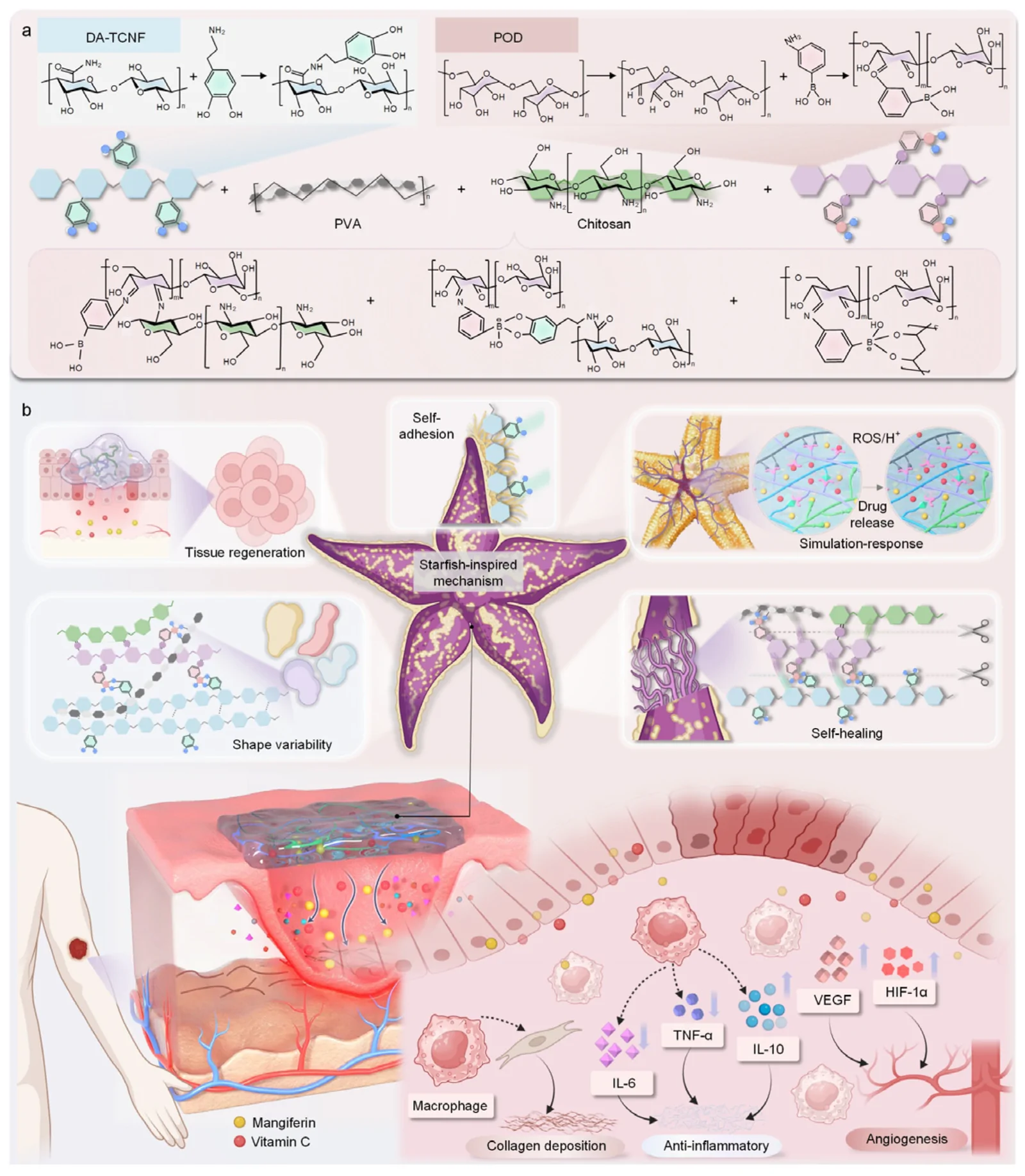
Design and multifunctional properties of the starfish-inspired bionic hydrogel. (**a**) Schematic illustration for hydrogel fabrication: dopamine-functionalized cellulose nanofibers (DA-CNFs), CS, BA-grafted oxidized dextran (BA-ODex) and PVA co-assemble into network via dynamic Schiff base and boronate ester bonds. (**b**) The biomimetic matrix possesses tissue adhesion, autonomous self-repair, shape conformability and tunable drug delivery capacity. It facilitates tissue reconstruction by curbing the secretion of pro-inflammatory TNF-α and IL-6, alongside elevated collagen deposition and angiogenic formation. Reproduced with permission from Ref. [80]. Copyright 2025, American Chemical Society.

Inspired by starfish, a bionically engineered hydrogel was synthesized through coassembly of dopamine (DA)-modified cellulose nanofibers, CS (3-aminobenzeneboronic acid)-grafted oxidized dextran, and poly (vinyl alcohol) utilizing dynamic Schiff base and boronic ester linkages. This hydrogel exhibited injectability, three-dimensional (3D) printability, antibacterial activity, self-adhesion, self-healing, antioxidant protection, and hemostasis, achieving complete wound closure by day 13 [80] (Figure 5).

##### Sponges and Films

Researchers blended Periplaneta americana remnant chitosan (PAC) and PAP derived from Periplaneta americana residues to fabricate a composite film designated PAPCF. According to relevant studies, this film possesses several valuable biological properties: it is compatible with body tissues, inhibits bacteria, relieves oxidative stress, and accelerates wound recovery [72]. In this composite film, PAC acts primarily as a structural scaffold with adjunctive antibacterial effects, whereas PAP serves as the principal immunomodulatory component driving the anti-inflammatory response.

Rapid hemostasis and clot formation were achieved when the sponge was applied to mouse liver injury models with heavy bleeding [81]. This multi-function porous scaffold is built through polyelectrolyte bonding of CS, SA and CMC, which endows the material with connected internal pores, good elasticity and fast liquid absorption capacity. Here, CS and its blends function predominantly as a structural scaffold for hemostasis and exudate absorption, with limited direct immunomodulatory contribution.

The three-dimensional hydrogel sponge based on CS and tannic acid (TA), modified with SA and arginine (Arg), can rapidly control bleeding at wound sites [82]. Besides its outstanding hemostatic performance, this sponge removes more than 90% of oxidative substances, holds plenty of water, and tolerates repeated compression without permanent deformation. In this sponge system, CS provides the structural framework for rapid hemostasis, while the incorporated TA and Arg primarily contribute antioxidant activity.

##### Nanofibrous Membranes and Microneedles

This double-layer nanofiber membrane, ENM, is made from two fiber layers produced by separate electrospinning steps. The bottom layer uses CS to carry zinc oxide nanoparticles, while the top layer is a mixture of PVA, CS and Ti_3_C_2_T_x_ MXene that wraps astaxanthin (AST). Researchers build this layered material by electrospinning each layer one after another. The double-layer nanofiber membrane can continuously deliver AST to lesion sites. The released compound clears redundant ROS and relieves inflammatory reactions, which collectively speeds up the repair of diabetic skin lesions [83].

Boronate ester bonds can trigger glucose-triggered substance release, and imine bonds are adopted to finely tune insulin release rates [84]. These two crosslinking structures are introduced into a biomimetic microneedle system that mimics pancreatic islets. The platform is constructed via joint modification of glycol, CS and dextran, and it delivers two major benefits: faster wound healing and long-lasting stable blood glucose management.

#### 3.2.2. Hyaluronic Acid

As a linear glycosaminoglycan without any side-chain substitutions, HA forms via repeated disaccharide motifs. These units alternate between β-1,3-linked D-glucuronic acid and β-1,4-connected N-acetyl-D-glucosamine. This biomolecule serves as one key constituent filling the ECM. Its molecular weight spans a wide range from several kDa to several MDa, and its immunomodulatory activity is strongly molecular weight-dependent: high-molecular-weight hyaluronic acid (HMW-HA, >1000 kDa) maintains tissue homeostasis, exerts anti-inflammatory effects and promotes M2 polarization, while low-molecular-weight hyaluronic acid (LMW-HA, <100 kDa) usually induces pro-inflammatory responses [85]. Hydrogel B exhibited superior resistance to degradation, enhanced mechanical properties, and reduced cytotoxicity in vitro. In this formulation, HA serves primarily as a structural scaffold, with the 4:1 HMW-HA/LMW-HA blend optimized for mechanical stability rather than direct immunomodulatory potency. This gel system is crosslinked via 1% BDDE (*v*/*v*), using 10% *w/v* HA raw material with HMW-HA and LMW-HA blended at a mass ratio of 4 to 1 [86].

Previous research reported a tunable injectable hydrogel scaffold optimized to remodel wound microenvironment, which was synthesized via two distinct conjugation routes.

**Figure 6:**
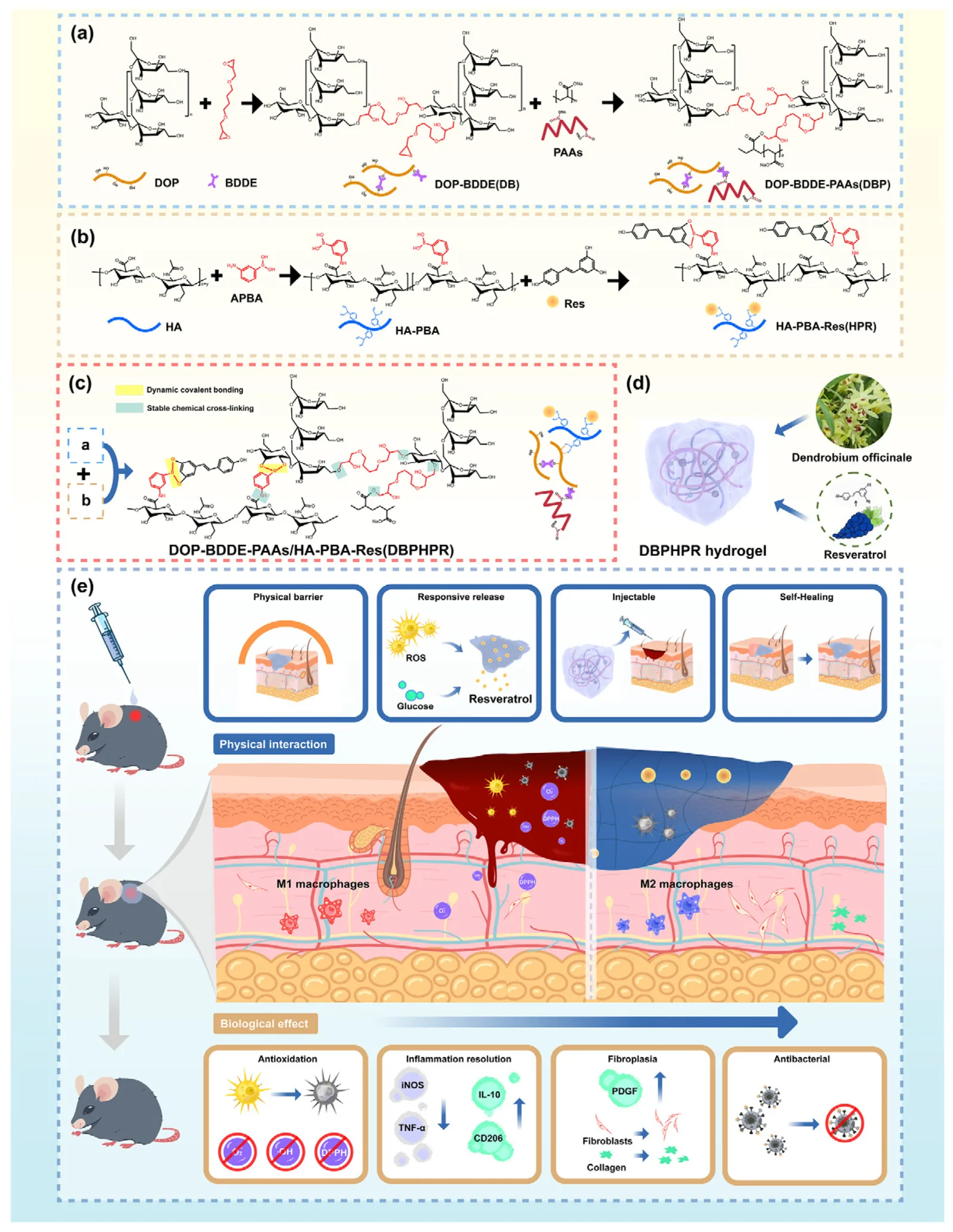
(**a**) DBP Synthesis Process: DOP, *Dendrobium officinale* polysaccharide; BDDE, 1,4-Butanediol diglycidyl ether; PAAs, sodium polyacrylate. (**b**) HPR Synthesis Process: HA, hyaluronic acid; APBA, 3-Aminophenylboronic acid; Res, resveratrol. (**c**,**d**) DBPHPR Hydrogel Synthesis Process. (**e**) Mechanism of DBPHPR Hydrogel in Promoting Wound Healing. Reproduced with permission from Ref. [87]. Copyright 2026, American Chemical Society.

In the first synthetic route, BDDE forms ether crosslinks with DOP and sodium polyacrylate (PAAS) to produce DBP intermediate. For the second branch, amide bonds anchor PBA onto HA, and boronate linkages further immobilize resveratrol (Res) on PBA to prepare HPR precursor. The final DBPHPR composite gel combines the two precursors and possesses favorable injectability and autonomous self-repair capacity. As documented in animal trials, the material releases Res upon exposure to elevated glucose or ROS, redirects macrophage differentiation toward an anti-inflammatory, M2-like phenotype, and accelerates tissue regeneration within full-thickness cutaneous lesions [87] (Figure 6). Here, DOP functions as the immunomodulatory agent driving M2 polarization, HA provides the structural scaffold with injectability, and PBA-Res conjugates serve as a glucose/ROS-responsive release vehicle.

To tackle diabetic skin lesions contaminated by bacteria, researchers constructed a multi-functional hydrogel matrix labeled HA-PBA/OAP/FeS@PC. Dual Schiff-base crosslinks are formed between HA-PBA and OAP to build the gel network, into which FeS nanoparticles modified with phycocyanin are embedded as functional fillers. This finished biomaterial features injectability, autonomous self-repair capacity and targeted responsiveness toward abnormal wound microenvironments [67]. This system has been detailed in Section 3.1.6 and is not repeated here.

An injectable self-healing HA-based hydrogel was developed based on multiple dynamic covalent bonds, exhibiting excellent injectable and self-healing properties, promoting axonal regeneration and functional recovery in traumatic spinal cord injury [88].

Three chemically modified biomacromolecular raw materials were selected as building blocks for HA composite hydrogel fabrication: aldehyde-grafted sodium hyaluronate (AHA), hydrazide-terminated sodium hyaluronate (ADA), and aldehyde-modified cellulose nanocrystals (oxi-CNC). Researchers combined the three components to assemble the target HA-based nanocomposite hydrogel. Dynamic acylhydrazone linkages drive instant in-situ gelation once the components are mixed, endowing the crosslinked network with boosted mechanical robustness and outstanding autonomous self-repair capacity. After incorporating platelet-rich plasma (PRP) into this matrix to produce ADAC@PRP dressing, the composite remarkably accelerates the recovery of full-thickness cutaneous wounds, as it facilitates granulation tissue growth, collagen accumulation and complete re-epithelialization of damaged skin [89].

To prepare wound dressings targeting oral mucosal ulcers, catechol-grafted sulfated hyaluronan (sHA-CA) was crosslinked together with catechol-functionalized high-molecular-weight hyaluronan (HMW-HA-CA) to form patch-type biomaterials. Animal trials on mice verified that these composite sHA-CA film dressings exert valid curative effects across three separate lesion models, including diabetic cutaneous injuries, liver bleeding defects, and postoperative tissue adhesion lesions [90].

#### 3.2.3. Sodium Alginate

Extracted from brown seaweed, SA is a naturally occurring anionic polysaccharide. Its long polymer backbone is constructed via α-1,4 glycosidic connections that join β-D-mannuronic acid (M) and α-L-guluronic acid (G) monomeric residues together. The molecular chain presents block distribution including poly-M, poly-G and MG alternating blocks. The molar ratio of M/G is generally 0.5–2.0 and varies with algal species; poly-G blocks can form rigid ionic crosslinks with divalent cations such as Ca^2+^, which endows alginate with excellent gel-forming properties. Although alginate contains mannuronic acid units, it belongs to uronic acid and has distinct properties from neutral mannose, thus classified as mannose-free polysaccharide. SA is widely used as an ideal carrier and skeleton material for composite functional hydrogels. It should be noted that SA itself has limited direct immunomodulatory activity, and is mainly used as an excellent scaffold carrier to synergize with bioactive polysaccharides or drug molecules.

##### Composite Hydrogels

Within the composite hydrogel platform consisting of OGLP, CMC and SA, SA acts as the core building unit that forms a dual crosslinked network. This structural design endows the whole scaffold with robust mechanical performance and favorable molding capacity. When combined with OGLP and CMC, SA exerts cooperative biological effects to shift macrophage differentiation from the pro-inflammatory M1 subtype toward anti-inflammatory M2 cells, which ultimately accelerates the repair of diabetic cutaneous lesions [57].

Mechanical testing revealed a 38% enhancement in tensile strength after forming dual crosslinked networks via hydrogen bonds and calcium ion interactions within hydrogel fibers. These reinforced SA and polyacrylate composite filaments are woven into wound-care textiles with directional programmable drug release capacity, and the resulting drug-incorporated fabric maintains air permeability and biosafety to support efficient skin injury therapy [91]. In this textile platform, SA acts solely as a structural scaffold, with its role limited to mechanical reinforcement and fiber formation rather than direct immunomodulation.

OSA crosslinked with CMCS forms a crosslinked gel matrix, which can encapsulate two therapeutic substances: astilbin liposomes (ALs) and diclofenac sodium (DS). This design yields a pH-responsive carrier capable of releasing two active payloads separately. Once this composite gel dressing covers diabetic skin lesions, it suppresses local inflammatory responses, accelerates new blood vessel formation, and stimulates fibroblast-mediated tissue reconstruction [92]. Here, OSA and CMCS serve as a pH-responsive delivery vehicle for the therapeutic payloads, with the polysaccharide matrix itself contributing limited direct immunomodulatory activity.

##### Films, Sponges, and Aerogels

The gelatin-SA matrix is blended with sea sheath and berberine hydrochloride to form the GS/CB composite membrane. Ionic crosslinkages are formed inside the polymer framework relying on Ca^2+^ originating from sea sheath components, which enhances tensile mechanical performance to 2.6 times the level of unmodified control films. Moreover, the membrane exhibited potent antibacterial activity against *Staphylococcus aureus* and MRSA [93].

Chlorogenic acid (CA) was incorporated into dopamine-functionalized alginate (Alg-DA) conjugates and utilized with PVA to fabricate multifunctional composite nanofiber membranes (PVA/Alg-DA/CA). These membranes exhibited high water absorption, porosity, and hydrophilicity, with excellent antibacterial and antioxidant activity, promoting neovascularization and tissue remodeling in a mouse full-thickness wound defect model [94]. In this nanofiber formulation, alginate provides the structural scaffold, while the incorporated CA contributes antioxidant and antibacterial functions.

A photothermal sponge wound patch, designated Alg-BA@PDA, was developed using BA-functionalized SA as the substrate and incorporates polydopamine nanoparticles as functional fillers. The sponge can absorb exudate rapidly and undergo on-site gel formation in merely 5 s, perfectly conforming to irregular wound surfaces. Biological characterizations verified that the composite sponge eliminates bacteria and clears redundant ROS; meanwhile, it facilitates the generation of new blood vessels and suppresses excessive inflammatory reactions to accelerate skin regeneration [95].

**Figure 7:**
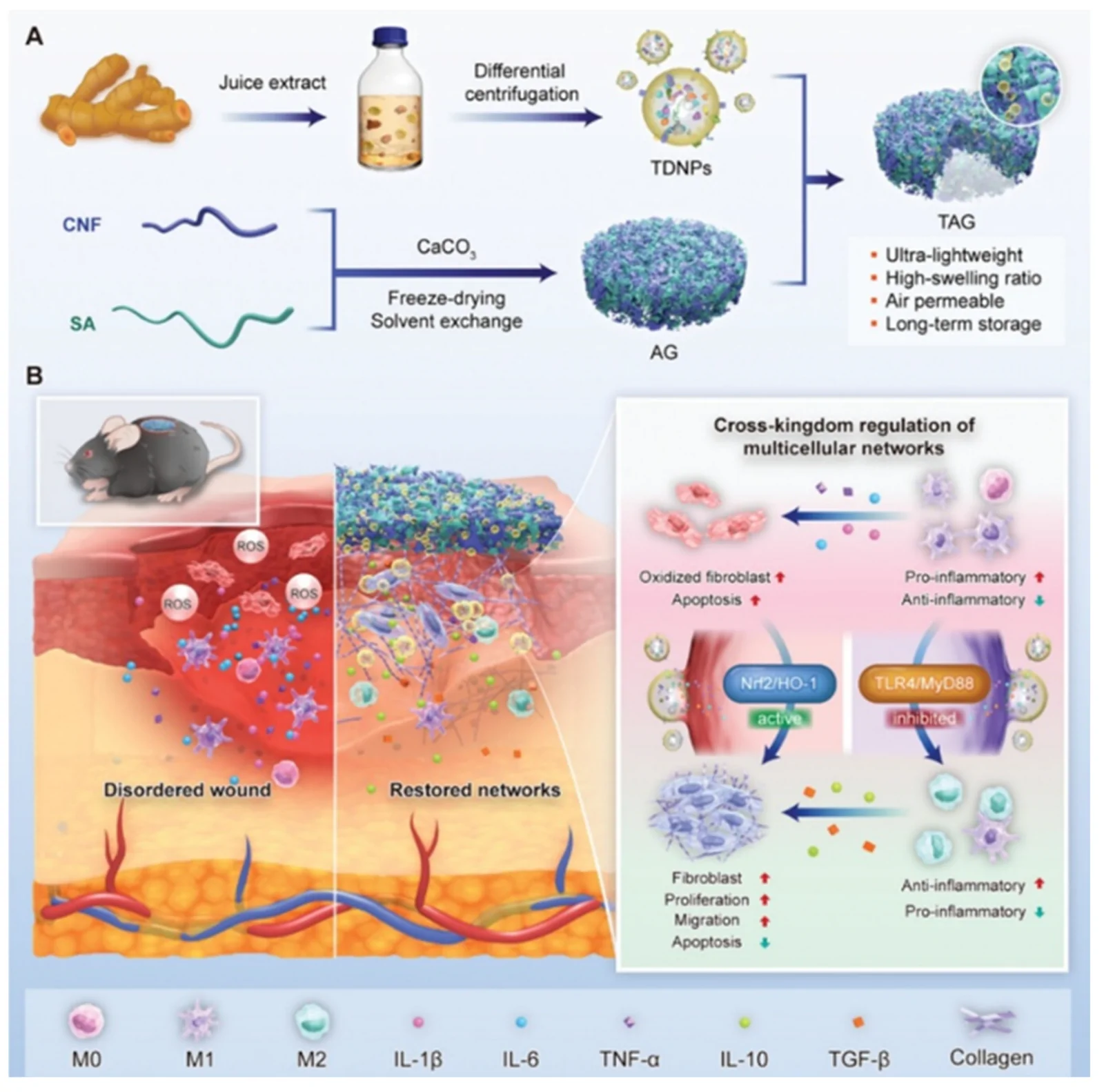
Schematic illustration of TDNP-incorporated aerogel (TAG) for diabetic cutaneous lesion repair through coordinated regulation of intercellular multicellular networks. (**A**) Isolation of TDNPs from turmeric juice and fabrication of TAG through freeze-drying of cellulose nanofibers (CNF) and SA scaffolds. (**B**) TAG modulates the wound microenvironment by scavenging ROS via the Nrf2/HO-1 pathway, inhibiting M1 macrophage polarization through the TLR4/MyD88 pathway, and promoting fibroblast proliferation and collagen deposition. TDNPs, turmeric-derived nanoparticles; AG, blank aerogel; CNF, cellulose nanofiber; SA; TAG, TDNP-loaded aerogel. Reprinted with permission from Ref. [96]. Licensed under CC BY 4.0.

Cellulose nanofibers and SA serve as dual skeletons to build an ultra-light, air-permeable aerogel carrier with powerful liquid absorption capacity, abbreviated as AG. Turmeric-origin nanoparticles (TDNPs) carrying natural bioactive ingredients are embedded within this porous scaffold to yield the TAG composite wound dressing. This aerogel composite can conform to wounds of arbitrary shapes, firmly adhere to moist lesion surfaces, and steadily deliver loaded TDNPs over a long period. Animal tests confirmed that the TAG system remarkably accelerates the regeneration of diabetic skin defects [96]. Here, SA and cellulose function as the dual structural scaffolds, whereas the turmeric-derived nanoparticles serve as the bioactive immunomodulatory and antioxidant components driving M2 polarization (Figure 7).

##### Microneedles and Microspheres

A multifunctional hyaluronic acid methacrylate (HAMA)/CMCS core–shell microneedle patch with SA as a moisture layer was developed. The microneedles stably adhered to wounds and penetrated biofilms, with the HAMA shell degraded by *Staphylococcus aureus*-secreted hyaluronidase, releasing graphene oxide with ASyycF to trigger antibacterial activity, while the CMCS core released basic fibroblast growth factor to accelerate angiogenesis and immune modulation [97].

Rod-like cellulose nanocrystals act as rigid skeleton fillers to fabricate sodium alginate composite microspheres (SA/CNC) through inverse emulsion fabrication. Afterwards, the cationic antibacterial macromolecule ε-polylysine undergoes spontaneous self-assembly onto the SA/CNC microsphere surface, which yields the final hemostatic porous microspheres abbreviated as PSLMs. This composite microsphere system possesses abundant internal void structures, powerful fluid uptake performance, outstanding blood clotting capacity and reliable bacteriostatic effects [98].

#### 3.2.4. Laminarin

Laminarin is a typical β-glucan derived from kelp, consisting primarily of β-D-glucopyranose units (>90%) linked by β-1,3 glycosidic bonds, with occasional β-1,6-linked short branches occurring approximately every ten residues along the main chain [99]. Although trace amounts of mannose may be present at the non-reducing ends of some samples, its monosaccharide composition is overwhelmingly glucose-dominated. Therefore, in accordance with the operational criteria established in Section 3.1 (mannose content < 20% and activity not mediated by mannose-specific receptors), laminarin is classified as mannose-free. Its primary immunomodulatory activity arises from the recognition of its β-glucan backbone by the C-type lectin receptor Dectin-1, which can trigger downstream signaling cascades including NF-κB [100]. However, as Dectin-1 recognizes β-glucan rather than mannose motifs, this receptor engagement does not qualify laminarin as a mannose-containing polysaccharide.

To date, the M2-polarizing activity of laminarin has been mainly validated in non-cutaneous models, such as intestinal inflammation and myocardial injury, where Dectin-1-mediated delivery of therapeutic payloads (e.g., miRNA-223) has been demonstrated [101]. Notably, its direct application in skin wound hydrogel dressings and its efficacy in cutaneous repair remain to be experimentally validated.

Conversely, polysaccharides such as laminarin, despite engaging C-type lectin receptors (e.g., Dectin-1), are classified as mannose-free because their immunomodulatory activity is driven by β-glucan recognition rather than mannose-specific receptor binding, underscoring that receptor family membership alone does not dictate categorical placement.

#### 3.2.5. *Lycium barbarum* Polysaccharide

*Lycium barbarum* L. berries are widely consumed as food and herbal medicine. As a well-documented medicinal plant in traditional Chinese medicine, this herb has been used for centuries to treat various ailments and support liver, kidney, and lung function. The primary bioactive substance extracted from Lycium barbarum is LBP, an acidic heteropolysaccharide that exists in glycoconjugate forms. Its molecular weight ranges from 10 kDa to 2300 kDa, and carbohydrate components make up more than 90% of its total mass. Four monosaccharides dominate the composition of LBP: arabinose, galactose, glucose and rhamnose. Mannose only takes up a small proportion, merely 5% to 10% of all sugar units contained within this polysaccharide. Galacturonic acid monomers are joined via α-1,4 connections to construct the core chain of this polysaccharide. Multiple other glycosidic bond configurations are also distributed across the molecule: α-(1→6)-Glc, β-(1→3)-Galp, β-(1→6)-Galp, α-(1→5)-Ara, alongside β-(1→4)-Galp linkages. The side chains are composed of arabinofuranose and galactopyranose residues, forming a highly branched pectin-like structure [102]. Lycium barbarum polysaccharide (LBP) delivers a broad spectrum of bioactive capacities, ranging from antioxidant, anti-inflammatory and immune-regulating effects to anti-tumor, neuroprotective and liver-protective functions. Its intricate branched molecular architecture allows this polysaccharide to simultaneously act on various inflammatory signaling axes and redirect macrophage polarization states. Meanwhile, the quality of harvested LBP varies drastically depending on which extraction workflow is adopted for sample isolation.

When spinal cord damage was induced in laboratory rats, LBP altered the polarization balance of macrophages and microglia: it suppressed M1 polarization while promoting an anti-inflammatory phenotype. This shift in immune cell phenotypes lessens excessive inflammatory reactions that arise after spinal cord trauma [103].

To solve the challenges of skin wound healing, researchers developed a combined treatment method. This approach combines electroacupuncture (EA) with conductive hydrogel microneedles made with polyphenols. Four raw materials make up this electrically conductive microneedle patch: methacrylated gelatin, DA, DA-grafted poly(3,4-ethylenedioxythiophene), and LBP. Stimulating the GV14 Dazhui acupoint via EA triggers the vagus-adrenal regulatory axis to attenuate systemic inflammatory responses. Meanwhile, DA-bound electric signals sustainably suppress inflammatory reactions right at the wound site. These two synergistic anti-inflammatory pathways together speed up the repair of diabetic skin lesions and relieve depression-related behavioral symptoms in animal models [104].

#### 3.2.6. Astragalus Polysaccharide

Astragali Radix contains a primary bioactive fraction known as APS, which belongs to the category of acidic heteropolysaccharides. It is mainly composed of glucose, galactose and arabinose with a molar ratio of approximately 5:3:2, and contains trace rhamnose and glucuronic acid. Its main chain consists of α-1,4-linked glucan and α-1,5-linked arabinan, with galactan side chains attached, forming a highly branched pectin-like structure [105]. Phenotypic conversion from a pro-inflammatory toward an anti-inflammatory macrophage state can be triggered by APS treatment in diabetic wound models [106]. At the molecular level, elevated β-catenin and Rspo3 alongside downregulated NF-κB and GSK-3β jointly mediate this immunological shift. In vivo animal assays further validated that this signaling cascade restrains late-stage inflammatory overreaction and facilitates the overall repair of diabetic wounds.

A traditional Chinese medicine microneedle patch was designed loaded with ROS-sensitive nanoparticles of APS, constructed into multifunctional composite microneedles (C/B@APB@Ber). The composite microneedle array can adhere stably to damaged skin tissue. Its robust mechanical properties enable the tip to penetrate bacterial biofilms. Meanwhile, the microneedle platform promotes cell proliferation and angiogenesis [107].

The 3D printing biomaterial loaded with APS can trigger nuclear translocation of STAT3 alongside activation of the YAP/TAZ signaling cascade. This cascade stimulation upregulates VEGF production within wound tissue, speeds up early-stage vascularization, and rectifies abnormal protein expression profiles to facilitate full-thickness cutaneous regeneration [108].

#### 3.2.7. Hylocereus Undatus Stem Polysaccharide

Hylocereus undatus stem polysaccharide (PSP) originates from byproducts of pitaya cultivation. It is a neutral heteropolysaccharide mainly composed of glucose, galactose and arabinose with a molar ratio of approximately 4:2:1, with a backbone of β-1,4-linked glucan and arabinogalactan side chains. PSP intervention elevated collagen deposition, suppressed pro-inflammatory cytokine release, and upregulated the angiogenic marker CD31 and the proliferation marker Ki67 in animal wound models. These phenotypic changes originate from PSP-induced macrophage polarization toward an anti-inflammatory, M2-like state, which substantially shortens the time required for complete wound closure in vivo. Single-cell RNA sequencing revealed that PSP upregulated genes associated with M2 polarization and may activate the PI3K/Akt signaling pathway [109].

#### 3.2.8. Concluding Remarks of Mannose-Free Polysaccharides

Compared with mannose-containing polysaccharides, mannose-free polysaccharides exhibit greater mechanistic diversity and more prominent material advantages in promoting anti-inflammatory macrophage polarization.

First, mechanistic diversity. These polysaccharides act through a variety of mechanisms, including cationic electrostatic interaction (CS), CD44 receptor recognition with molecular weight dependence (HA), ionic cross-linking and carrier-assisted synergy (alginate), and multi-targeted signaling regulation (APS, pitaya stem polysaccharide). Unlike mannose-containing polysaccharides that mostly act through direct receptor recognition, mannose-free polysaccharides often combine direct immunomodulation with indirect anti-inflammatory effects (antibacterial, ROS scavenging), providing abundant options for the design of multifunctional composite hydrogels.

Second, outstanding material advantages. The cationic and film-forming properties of CS, the cytocompatibility of HA, and the mild cross-linking conditions of alginate make these polysaccharides uniquely advantageous for constructing hydrogels with good mechanical properties and clinical adaptability.

Third, composite synergy is the mainstream strategy. Since the immunomodulatory activity of a single mannose-free polysaccharide is often limited, current research mostly adopts a composite strategy, using such polysaccharides as backbone materials and combining them with more active mannose-containing polysaccharides or drug molecules to achieve synergistic optimization of immunomodulatory functions and material properties.

The majority of published works only dissect the classic NF-κB and PI3K/Akt signaling cascades to interpret polysaccharide immune regulation, leaving many unknown molecular mechanisms waiting to be clarified. Another prominent research gap lies in metabolic reprogramming studies: mannose-containing polysaccharides dominate relevant reports, whereas mannose-free counterparts rarely receive attention. The key structural features, receptor targets, signaling pathways, and material roles of representative pro-M2 natural polysaccharides are summarized in Table 1.

### 3.3. Functional Modification Strategies of Natural Polysaccharides and Their Immunomodulatory Consequences

Natural polysaccharides often suffer from limited solubility, weak mechanical strength, rapid enzymatic degradation, and poor controlled-release capacity, necessitating chemical functionalization to render them clinically viable. Chemical functionalization has therefore emerged as an indispensable strategy to tailor their performance profiles. Critically, such modifications are not merely structural adjustments; they profoundly alter molecular recognition patterns, charge distributions, and receptor accessibility, thereby directly or indirectly modulating immunomodulatory potency and macrophage polarization outcomes.

Oxidation and Aldehyde Generation. Periodate oxidation represents one of the most widely adopted strategies to introduce reactive aldehyde groups along the polysaccharide backbone, enabling subsequent crosslinking via Schiff-base or acylhydrazone chemistry. For instance, oxidation of GLP generates dialdehyde functionalities that react with amine-bearing counterparts such as CMCS, yielding injectable, self-healing hydrogels with tunable degradation kinetics. Similarly, OSA and OAP have been employed as dynamic crosslinkers in multifunctional dressings. From an immunomodulatory perspective, moderate oxidation preserves native β-glucan or mannan structural motifs required for Dectin-1 or MR engagement, whereas excessive oxidation can disrupt these recognition epitopes and generate cytotoxic by-products that paradoxically trigger pro-inflammatory stress responses.

Carboxymethylation and Sulfation. Carboxymethylation introduces carboxymethyl (–CH_2_COOH) substituents, substantially enhancing water solubility and anionic charge density under physiological pH. CMCS and CMC exemplify this approach. The increased negative charge facilitates electrostatic interactions with cationic immune cell membranes and promotes the formation of polyelectrolyte complexes. Sulfation, though less frequently reported in the cutaneous wound-healing literature, generates highly anionic sulfate esters that can mimic glycosaminoglycans such as heparan sulfate, potentially engaging scavenger receptors and modulating NF-κB signaling. Nevertheless, the immunological consequences of these charge modifications are context-dependent; while carboxymethylation generally improves cytocompatibility and scaffold integration, excessively high substitution degrees may nonspecifically adsorb serum proteins and obscure the polysaccharide’s intrinsic receptor–ligand interactions.

Deacetylation and Quaternization. For CS, the DDA is a critical structural parameter governing biological activity. Higher DDA (>85%) yields greater densities of free amino groups, enhancing cationic character, mucoadhesion, and antibacterial efficacy through membrane disruption. Concurrently, this elevated positive charge density has been associated with suppression of TLR4-driven NF-κB activation and promotion of M2-like polarization in LPS-stimulated macrophages. Quaternization—typically via grafting of trimethylammonium or guanidinium moieties—further amplifies these effects by conferring permanent positive charges independent of ambient pH. Nevertheless, excessive cationic strength can induce erythrocyte aggregation and pro-inflammatory cytokine release, necessitating careful optimization of the DDA or quaternization degree to balance antibacterial activity with anti-inflammatory macrophage reprogramming.

Grafting of Bioactive Functional Moieties. Surface grafting with small-molecule or oligomeric ligands enables the integration of secondary biological functionalities without sacrificing the primary polysaccharide scaffold. Representative examples include DA grafting (imparting tissue adhesion and antioxidant capacity via catechol chemistry), PBA conjugation (enabling glucose-responsive drug release through reversible boronate ester formation), and ferulic acid coupling (augmenting ROS-scavenging capacity). These functional groups can indirectly influence macrophage polarization by neutralizing oxidative stress, modulating local glucose concentrations, or releasing bound anti-inflammatory payloads in a spatiotemporally controlled manner. However, grafting density and linker chemistry must be optimized to prevent steric hindrance of the polysaccharide’s native recognition motifs.

Hybridization with Synthetic Polymers and Inorganic Nanoparticles. Blending natural polysaccharides with synthetic polymers (e.g., PVA, polyacrylate) or incorporating metallic/metalloid nanoparticles (e.g., AgNPs, ZnO, FeS) is frequently employed to reinforce mechanical properties, introduce photothermal or antibacterial functions, and create stimuli-responsive systems. However, the specific contribution of the polysaccharide to M2 polarization must be distinguished from the effects of synthetic or inorganic components. For example, silver nanoparticles possess intrinsic anti-inflammatory properties that may synergize with, or mask, the polysaccharide’s immunomodulatory signaling. Therefore, mechanistic studies on composite systems should include polysaccharide-only controls to disambiguate scaffold, carrier, and immunomodulatory roles.

Concluding Remarks on Functionalization. Collectively, functional modification serves as a powerful translational bridge between raw natural polysaccharides and clinically viable wound dressings. A key challenge for future research is to systematically correlate the type, density, and position of chemical modifications with quantitative macrophage phenotypic readouts, moving beyond empirical formulation toward rational, mechanism-guided design.

### 3.4. Advanced Biomaterials Design Strategies for Polysaccharide Hydrogels

Building on the systematic overview of source structures, immunomodulatory mechanisms, and dressing constructions of representative polysaccharides in the previous two sections, this section further summarizes advanced construction strategies for polysaccharide hydrogels, focusing on biochemical–physical synergistic regulation and application-driven dosage form design.

#### 3.4.1. Synergistic “Biochemical–Physical” Regulation of Polysaccharide Hydrogels

Chemical ligand recognition alone may be insufficient to reverse established M1 polarization in chronic wounds, and modern hydrogel design is moving towards “biochemical–physical” coupling.

Mechanical guidance via viscoelasticity and stiffness

Macrophages are highly sensitive to the mechanical properties of the substrate. Excessively stiff substrates (>100 kPa) exacerbate M1 pro-inflammatory polarization via YAP/TAZ nuclear translocation, whereas softer polysaccharide hydrogels with stress-relaxation properties (e.g., alginate-gelatin double-network gels, 1–10 kPa) facilitate macrophage elongation and anti-inflammatory factor secretion.

Notably, the effect of substrate stiffness on macrophage polarization remains controversial in the field. Inconsistent findings have been reported regarding whether moderate stiffness promotes or inhibits inflammation, which may be attributed to differences in cell sources (murine cell lines vs. primary human macrophages), stiffness ranges, surface chemistry, protein adsorption and viscoelastic properties of substrates. Clear human cellular evidence for mechanical stiffness-mediated immune regulation can be obtained from experiments carried out on alginate methacrylate hydrogels with human monocyte-derived macrophages [110]. Within the biomechanically relevant stiffness window of soft tissues (0.25–4.5 kPa), pro-inflammatory biomarkers like TNF-α are continuously overexpressed as substrate rigidity rises, whereas milder, low-stiffness matrices facilitate anti-inflammatory M2 macrophage polarization. These findings suggest that the immunomodulatory effect of stiffness is highly context-dependent, and its translation to wound dressing design requires comprehensive consideration of the in vivo microenvironment.

Topology and spatial confinement

Polysaccharide scaffolds constructed by combining 3D printing and electrospinning technologies have micropore sizes that directly affect macrophage morphology. Smaller pore sizes or microgroove structures can force macrophages to elongate, and this physical deformation of the cytoskeleton can alter the epigenetic modification of chromatin through the linker of nucleoskeleton and cytoskeleton (LINC) complex, thus directly driving macrophage polarization toward the pro-angiogenic M2 phenotype. In collagen-CS hybrid hydrogel systems, aligned fibrous topologies fabricated via plastic compression have also been demonstrated to drive macrophage polarization toward the M2 phenotype and orchestrate the sequential transition from myofibroblast-mediated wound contraction to regenerative tissue remodeling, ultimately achieving scarless wound repair with complete re-epithelialization within 7 days in a rat full-thickness skin defect model.

Cascade release with spatiotemporal delivery

Given that normal wound healing requires a transition from a “transient inflammatory phase” to a “proliferative reparative phase,” promoting M2 polarization too early may lead to uncontrolled infection. Ideally, polysaccharide hydrogels should have the ability to control release timing: rapid degradation of the outer layer to release antimicrobial/pro-inflammatory signals (clearing infection), responsive degradation of the inner core by MMPs or ROS, and slow release of mannose-containing polysaccharides or metabolism-regulating molecules in the late inflammatory phase, precisely matching the physiological timeline of wound healing.

#### 3.4.2. Design Strategies by Dosage Form

The performance of polysaccharide hydrogels is highly dependent on the matching between dosage form and clinical scenario. Different wound types have distinct therapeutic demands, and targeted dosage form design can maximize immunomodulatory efficacy. Based on existing research, the general matching principles are summarized as follows:

Acute wounds (burns, surgical incisions, trauma): Core demands are rapid hemostasis, antimicrobial activity and early inflammation modulation. Porous sponges and films based on CS or alginate are preferred: CS-based materials exert rapid hemostatic and antibacterial effects through cationic action, while alginate-based materials can absorb a large amount of exudate via ionic gelation, which are suitable for acute wounds with high exudate volume.

Chronic wounds (diabetic foot ulcers, pressure ulcers): Core demands are sustained immunomodulation, antioxidant stress and anti-biofilm activity. Injectable self-healing hydrogels (e.g., Schiff base crosslinked CS/HA systems) and microneedle arrays are particularly suitable dosage forms. Injectable hydrogels can adapt to irregular wound beds and realize spatiotemporally controlled release of M2-promoting polysaccharides; microneedle arrays can penetrate the wound biofilm barrier and deliver active ingredients directly to deep wound tissues, avoiding premature degradation in the superficial exudate.

Full-thickness skin defects and tissue engineering: Core demands are structural support, cell infiltration and sequential tissue regeneration. 3D-printed polysaccharide scaffolds with gradient pore structure are suitable, which can mimic the layered structure of natural skin. The combination with conductive or piezoelectric components can further promote cell migration and M2 polarization through electrical-mechanical synergy, so as to orchestrate the whole process of wound repair.

## 4. Conclusions and Outlook

### 4.1. Summary

Natural polysaccharides have transitioned from serving merely as structural scaffolds to functioning as bioactive macromolecules that actively orchestrate the wound immune microenvironment. Their ability to modulate macrophage polarization—particularly toward the anti-inflammatory and pro-reparative M2 phenotype—represents an evolving strategy in wound dressing design, moving from passive coverage to active immunoregulation. The mechanistic basis for this activity lies in the recognition of specific polysaccharide structural motifs by macrophage surface receptors (e.g., MR, Dectin-1, CD44), which trigger downstream signaling cascades (NF-κB, STAT6, PI3K/Akt) and metabolic reprogramming (e.g., glycolysis inhibition, O-GlcNAcylation). The Structural features, receptor targets, signaling pathways, and material roles of representative pro-M2 natural polysaccharides are summarized in Table 1 for quick reference and comparative analysis. The corresponding preclinical evidence, including dosage forms, experimental models, macrophage markers, evidence levels, and therapeutic outcomes, is summarized in Table 2. Two functional subgroups are defined for these polysaccharides based on variations in their monosaccharide components. Polysaccharides integrated with mannose motifs are primarily recognized by C-type lectin receptors, whereas mannose-free counterparts trigger cellular responses through cationic interactions, CD44 ligation, or simultaneous activation of multiple target signaling axes. Nevertheless, it must be emphasized that the majority of these encouraging outcomes originate from well-controlled animal studies, and their direct translatability to the heterogeneous clinical setting—particularly in patients with diverse comorbidities, medication profiles, and wound aetiologies—remains to be rigorously established. Therefore, while the immunomodulatory paradigm offered by polysaccharide hydrogels is conceptually appealing, current evidence should be interpreted as proof-of-concept rather than as conclusive clinical validation.

### 4.2. Challenges and Translational Bottlenecks

#### 4.2.1. Intrinsic Limitations of Natural Polysaccharides in Cutaneous Wound Healing

Before addressing the extrinsic challenges of device fabrication and clinical translation, it is essential to acknowledge the inherent limitations of natural polysaccharides as macromolecular starting materials for cutaneous wound management. These limitations stem primarily from their biological origin and structural complexity, and they fundamentally constrain the reproducibility, predictability, and standalone efficacy of polysaccharide-based dressings.

Structural Heterogeneity and Batch-to-Batch Variability. Unlike synthetic polymers with precisely defined repeat units and monodisperse molecular weights, natural polysaccharides are inherently heterogeneous. Parameters such as molecular weight distribution, branching degree, monosaccharide ratios, and glycosidic linkage patterns vary markedly with species, origin, cultivation conditions, harvest season, and extraction protocol. For example, the mannose-to-glucose ratio in KGM fluctuates between 1.5:1 and 2.0:1 across different cultivars, while the degree of acetylation in DOP is highly sensitive to extraction temperature and solvent polarity. This variability introduces substantial uncertainty into immunomodulatory outcomes, as receptor binding affinity and downstream signaling potency are exquisitely sensitive to these fine structural details. Without rigorous standardization and multivariate quality control, reproducible therapeutic efficacy cannot be guaranteed.

Extraction-Derived Contaminants. Raw polysaccharide isolates frequently carry co-extracted impurities, including endotoxins (lipopolysaccharides), residual proteins, polyphenols, and heavy metal ions. Endotoxin contamination is particularly problematic, as nanogram quantities can activate TLR4/NF-κB signaling and produce artifactual pro-inflammatory responses that may be wrongly attributed to the polysaccharide. Similarly, residual proteins or phenolic compounds can engage scavenger receptors or modulate oxidative stress pathways, confounding the interpretation of structure–activity relationships. Current purification protocols lack universal standards, and many studies fail to report endotoxin levels or residual protein content, undermining the mechanistic validity of reported M2-polarizing effects.

Physicochemical Constraints. Many native polysaccharides exhibit physicochemical properties that limit their standalone utility as wound dressings. CS, for instance, is soluble only in acidic aqueous media, restricting its direct application to neutral pH wound beds without chemical modification. SA forms hydrogels rapidly in the presence of divalent cations but suffers from rapid dissolution and loss of structural integrity under high-exudate conditions. Furthermore, the mechanical strength of pure polysaccharide hydrogels is often orders of magnitude lower than that of native skin ECM, potentially compromising their ability to protect the wound bed or support cellular infiltration. The degradation kinetics of native polysaccharides are also difficult to synchronize with the temporal phases of wound healing; overly rapid degradation may deprive the wound of sustained immunomodulatory signaling during the proliferative phase, whereas overly slow degradation may physically impede tissue remodeling.

Unpredictable Interactions with the Wound Microenvironment. The cutaneous wound microenvironment is characterized by dynamic fluctuations in pH (typically 5.5–8.5), elevated ROS, proteolytic enzymes (e.g., MMPs), and polymicrobial biofilms. Native polysaccharides may undergo non-specific enzymatic cleavage, oxidative depolymerization, or electrostatic complexation with wound exudate proteins, leading to uncontrolled alterations in their effective concentration, molecular weight, and bioactivity. Additionally, some polysaccharides may inadvertently serve as nutrient sources for wound-colonizing bacteria, potentially exacerbating infection. These unpredictable interactions underscore the necessity of protective formulation strategies—such as chemical crosslinking, encapsulation, or composite engineering—to stabilize polysaccharide bioactivity in situ.

Recognizing these intrinsic limitations is not merely an academic exercise. It provides the fundamental rationale for the functional modification strategies and composite formulations discussed above, and underscores the urgent need for standardized extraction, characterization, and quality control protocols that are currently lacking in the field.

#### 4.2.2. Translational Bottlenecks: From Preclinical Promise to Clinical Reality

Beyond the intrinsic limitations discussed above, translating polysaccharide-based hydrogels from preclinical studies to routine clinical practice faces several interconnected hurdles that are often underestimated in the materials science literature. First, the majority of available evidence derives from rodent models, which differ substantially from human skin in terms of thickness, hair follicle density, wound contraction mechanisms (predominantly mediated by the panniculus carnosus in mice versus granulation tissue in humans), and immune system composition. These anatomical and physiological disparities may compromise the predictive validity of preclinical efficacy data, as a dressing that achieves near-complete closure in a murine diabetic wound within 14 days may elicit a considerably more modest response in a human patient with peripheral arterial disease, neuropathy, and chronic biofilm colonization. Consequently, therapeutic effects observed in animal studies should be interpreted as proof-of-concept rather than as reliable predictors of clinical performance.

Second, the complexity of human chronic wounds—characterized by polymicrobial infections, protease-rich exudate, hypoxia, and systemic comorbidities such as renal impairment or immunosuppression—is rarely recapitulated in standard preclinical models. The discrepancy between well-controlled laboratory conditions and the heterogeneous, dynamic microenvironment of real-world ulcers poses a substantial challenge for the reproducibility and consistency of hydrogel-mediated immunomodulation. Third, regulatory pathways for hydrogel-based wound dressings remain ambiguous, as these products may be classified as medical devices, drug–device combinations, or even biologics depending on their composition and mechanism of action. This classification uncertainty complicates the design of clinical trials, lengthens approval timelines, and increases development costs. Fourth, the scalability and batch-to-batch reproducibility of natural polysaccharide hydrogels—issues intimately connected to the raw-material variability discussed earlier—remain unresolved for many formulations, hindering the transition from laboratory-scale fabrication to Good Manufacturing Practice (GMP)-compliant industrial production. Finally, the lack of standardized clinical endpoints and validated biomarkers for assessing macrophage polarization in human wound tissue impedes the objective evaluation of immunomodulatory efficacy in early-phase trials. Addressing these translational bottlenecks will require interdisciplinary collaboration among material scientists, immunologists, wound-care clinicians, and regulatory experts, as well as the establishment of large-scale, prospective clinical registries to capture real-world performance data. Only through such concerted efforts can the gap between bench innovation and bedside application be meaningfully narrowed.

### 4.3. Future Prospects

First, standardized material systems and modular functional platforms. The extraction, purification, modification, and characterization processes for natural polysaccharides require standardization to build reliable raw material libraries and quality control systems. Building on this foundation, modular functional unit libraries (e.g., integrating antimicrobial, M2 polarization, and angiogenic modules) should be developed. Adopting a “plug-and-play” strategy will facilitate the rapid assembly and performance optimization of these multifunctional hydrogels.

Second, integrating advanced manufacturing technologies to build a personalized treatment platform. By combining microfluidic, three-dimensional (3D) printing and bio-ink technologies, programmable and customizable smart hydrogel dressings can be developed to achieve precise adaptation of complex wound morphology and personalized regulation of patient-specific therapeutic dosage and release timing. Meanwhile, minimally invasive application forms such as sprayable and injectable are explored to enhance the convenience of clinical operation.

Third, the molecular mechanisms of material–host interactions should be systematically elucidated. With the help of multi-omics technologies (single-cell transcriptomics, metabolomics, proteomics), organoid models and high-resolution in situ imaging, we systematically elucidate the key molecular events and signaling networks of polysaccharide hydrogels in immune cell recruitment, metabolic reprogramming, phenotypic transformation and tissue regeneration, and reveal the intrinsic correlation of structure–activity-mechanism, providing theoretical guidance for targeted screening and rational design of highly active polysaccharides.

Fourth, artificial intelligence (AI)-assisted rational design: machine learning algorithms could be trained. Machine learning algorithms could be trained on existing SAR data of natural polysaccharides to predict novel polysaccharide sequences, modification patterns or monosaccharide ratios with optimal M2-polarizing activity, which can greatly reduce the workload of experimental screening and improve the efficiency of material development.

Fifth, engineered living materials. Engineered living hydrogels that encapsulate probiotic bacteria capable of producing M2-polarizing polysaccharides or metabolites directly at the wound site represent an emerging and promising avenue. These biomaterial platforms adjust their functional behaviors in real time to match shifting local conditions within wound tissue. Meanwhile, they are capable of delivering long-lasting immune regulation that activates only when the lesion site requires therapeutic intervention.

Sixth, closed-loop feedback intelligent systems. Integrating biosensors (e.g., for glucose, pH, or MMPs) into the hydrogel to enable closed-loop, on-demand delivery of immunomodulatory signals is the ultimate goal for personalized wound care. This “sensing-diagnosis-treatment” integrated system can realize real-time dynamic response to the wound state and provide precise treatment solutions for individual patients.

The cumulative evidence reviewed herein suggests that natural polysaccharide hydrogels are emerging as a versatile and biologically interactive platform in the field of skin regeneration, primarily by virtue of their intrinsic immunomodulatory properties and favorable physicochemical characteristics. Nevertheless, the trajectory from laboratory innovation to clinical standard-of-care is contingent upon continued progress in several interconnected domains: the establishment of standardized manufacturing protocols, the elucidation of structure–activity relationships through systematic comparative studies, and the execution of well-powered, multi-centre clinical trials that incorporate patient-relevant endpoints. If these hurdles can be addressed, such biomaterial systems may eventually transcend the role of passive physical barriers, evolving into ‘smart’ therapeutic constructs that actively sense and remodel the wound microenvironment. Ultimately, this evolution holds the potential—though not yet the certainty—to offer safer and more effective alternatives for managing chronic, non-healing wounds, provided that rigorous safety and efficacy benchmarks are met in future translational investigations.

**Table 1:** Structural features, receptor targets, signaling pathways, and material roles of representative pro-M2 natural polysaccharides.

Category	Polysaccharide	Main Monosaccharide Composition	Backbone Linkage	Primary Receptor (s)	Key Signaling Pathway (s)	Polysaccharide Role	Refs
Man-nose-containing	KGM	Mannose:Glucose ≈ 1.6:1	β-1,4 glycosidic; minor β-1,3/β-1,6 branching	SIGNR1 (direct: KO mice); MR (structurally inferred)	c-Raf/NF-κB; TNF-α/NF-κB inhibition	Immunomodulator + scaffold	[47,48,49,50,51,52]
Man-nose-containing	BSP	Mannose:Glucose ≈ 3:1	β-1,4 glycosidic; C-3 branching	Structurally inferred (MR/TLR4); no direct validation	NF-κB inhibition	Immunomodulator + scaffold	[53,54,55]
Man-nose-containing	GLP	Glucose 60–70%; Mannose 10–20%	β-1,3 glucan backbone; β-1,6 branching	Structurally inferred (Dectin-1/MR); no direct binding assays reported	NF-κB inhibition (p-p65/p65 expression); ROS scavenging	Immunomodulator	[56,57,58,59,60]
Man-nose-containing	DOP	Mannose:Glucose ≈ 3:1–5:1	β-1,4 glycosidic; C-2/C-3 acetylation	Structurally inferred (MR/SIGNR1); no direct receptor validation	SENP1-SIRT3 axis (non-cutaneous); AKT pathway	Immunomodulator	[61,62,63]
Man-nose-containing	Aloe polysaccharide	Mannose > 70%	β-1,4 mannan backbone; C-6 acetylation	Not identified (anti-inflammatory phenotype observed)	NF-κB inhibition	Immunomodulator + scaffold	[64,66,68]
Man-nose-containing	*Anoectochilus xingrenensis* X21	Gal:Glc:Man ≈ 2.5:1.8:1	→4)-β-D-Galp-(1→, →4)-α-D-Glcp-(1→, →4,6)-α-D-Glcp-(1→	Not identified	NF-κB/MAPK inhibition; STAT6 activation (non-cutaneous)	Immunomodulator (mechanistic corroboration only; non-cutaneous)	[69]
Man-nose-containing	*Periplaneta americana* PAP55-3-1	Glc:Gal:Ara:Man ≈ 5.2:2.8:1.35:6.5	α-1,4-Glc; β-1,3-Gal	Not identified	O-GlcNAc-HIF-1α/PPARγ pathway	Immunomodulator (in CS composite film)	[70,71,72]
Man-nose-containing	*Schizophyllum commune* F2	Mannose 55.2 mol%; Glucose 28.7 mol%	(1→3)-mannan backbone; (1→2,3)-branching	CR-3, TLR-4 (indirect: inferred from cytokine profile)	NF-κB/MAPK activation (non-cutaneous; in vitro)	Mechanistic corroboration only (non-cutaneous; in vitro)	[73,74]
Mannose-free	CS	Glucosamine:N-acetylglucosamine ≈ 7:3–9.5:0.5	β-1,4 glycosidic	TLR4 (indirect: inhibitor studies); other receptors may contribute	NF-κB inhibition; ROS scavenging	Scaffold + antibacterial + carrier	[77,78,79,80,81,82,83,84]
Mannose-free	HA	Glucuronic acid:N-acetylglucosamine = 1:1	Alternating β-1,3/β-1,4 glycosidic	CD44 (established in the foundational literature; not directly validated in cited wound studies)	PI3K/Akt, STAT6 (inferred)	Scaffold + carrier	[85,87,89,90]
Mannose-free	SA	Mannuronic acid:Guluronic acid ≈ 0.5–2.0	α-1,4 block-type glycosidic	Non-specific (primarily carrier)	Synergistic with active components	Scaffold (primary) + carrier	[91,92,93,94,95,96,97,98]
Mannose-free	Laminarin	Glucose > 90%	β-1,3 glucan backbone; β-1,6 branching	Dectin-1 (binding established for β-glucans in the foundational literature; Laminarin-specific M2 activity not skin-validated)	NF-κB (inferred)	Carrier (mechanistic corroboration only; non-cutaneous)	[99,100,101]
Mannose-free	LBP	Arabinose, Galactose, Glucose, Rhamnose; Mannose 5–10%	α-1,4-GalpA backbone; multiple branch linkages	Not identified	M1/M2 phenotype shift (non-cutaneous); not validated in wound model	Functional additive (role not independently validated)	[102,103,104]
Mannose-free	APS	Glucose:Galactose:Arabinose ≈ 5:3:2	α-1,4 glucan + α-1,5 arabinan	Not identified (phenotype only; β-catenin/NF-κB validated)	β-catenin/NF-κB axis; YAP/TAZ	Immunomodulator	[105,106,107,108]
Mannose-free	PSP	Glucose:Galactose:Arabinose ≈ 4:2:1	β-1,4 glucan backbone; arabinogalactan side chains	Not identified	PI3K/Akt (scRNA-seq correlation only)	Immunomodulator	[109]

**Table 2:** Preclinical evaluation: dosage forms, experimental models, macrophage markers, evidence levels, and therapeutic outcomes (↑: upregulation; ↓: downregulation).

Category	Polysaccharide	Typical Dosage Form (s)	Experimental Model (s)	Macrophage Markers Examined	Evidence Level for Receptor/Pathway	Representative Therapeutic Effect (s)	Refs
Mannose-containing	KGM	Composite hydrogel; sponge; fibrous membrane	In vitro: RAW264.7; In vivo: mouse full-thickness, mouse diabetic; non-cutaneous mechanistic: intestinal inflammation (SIGNR1 KO)	↑CD206, IL-10; ↓TNF-α	Direct for SIGNR1 in non-cutaneous model: KO mice [49]; Indirect for NF-κB: inhibitor studies [50]; MR: structurally inferred	re-epithelialisation; collagen deposition	[49,50,51,52]
Mannose-containing	BSP	Self-healing hydrogel; blended hydrogel	In vivo: mouse diabetic ulcer, rat full-thickness	↑CD206, IL-10; ↓iNOS	Indirect: NF-κB inhibition via inhibitor studies [54]	94% wound closure; >90% antibacterial rate	[54,55]
Mannose-containing	GLP	Double-network hydrogel; thermosensitive hydrogel; yolk–shell particles	In vitro: RAW264.7; In vivo: mouse diabetic, burn wound	↑CD206, Arg-1; ↓CD86, iNOS	Indirect for NF-κB: inferred from p-p65/p65 expression [57]; Indirect for Dectin-1/MR: structurally inferred; no direct binding or inhibitor studies reported	M2 polarisation; diabetic wound healing; photothermal/antibacterial effects	[56,57,58,59,60]
Mannose-containing	DOP	Ionic composite hydrogel	In vitro: BMDMs [62]; In vivo: mouse diabetic [63]; non-cutaneous mechanistic: colitis model [62]	↑CD206, Arg-1, IL-10; ↓TNF-α, IL-1β, IL-6, iNOS	Indirect for SENP1-SIRT3: inferred from protein expression and siRNA validation [62]; Indirect for AKT: pathway inhibitors [63]; MR/SIGNR1: structurally inferred	Anti-inflammatory; antioxidant; angiogenesis; M2 polarisation	[61,62,63]
Mannose-containing	Aloe polysaccharide	Porous dressing; responsive hydrogel; nanofiber	In vivo: mouse full-thickness, mouse infected diabetic	↑CD206, IL-10; ↓TNF-α, CD86	Indirect: NF-κB inhibition via inhibitor studies [66]; receptor not identified	Suppressed inflammation; angiogenesis; antibacterial	[66,67,68]
Mannose-containing	X21	Not reported for skin	In vivo: rat collagen-induced arthritis (non-cutaneous; mechanistic only)	Not examined in cutaneous context	Indirect: NF-κB/MAPK inhibition; STAT6 activation via inhibitor studies [69]	Anti-arthritic effects; no skin data reported	[69]
Mannose-containing	PAP55-3-1	PAPCF	In vitro: RAW264.7; In vivo: mouse cutaneous wound	PPARγ↑ examined in vitro [71]; classical M2 markers not specified in wound model [72]	Indirect: O-GlcNAc-HIF-1α/PPARγ via metabolic modulation [71]	Wound contraction; collagen deposition	[70,71,72]
Mannose-containing	S. commune F2	Not reported for skin	In vitro: RAW264.7 (non-cutaneous; mechanistic only)	NO and cytokine secretion measured	Indirect: CR-3 and TLR-4 involvement hypothesized from cytokine profile; no blocking or KO validation reported [74]	NO/cytokine secretion; no skin data reported	[73,74]
Mannose-free	CS	Self-healing hydrogel; self-healing hydrogel; sponge; nanofiber; microneedle; biomimetic hydrogel	In vitro: macrophages; In vivo: rat infected, mouse diabetic	↑CD206, IL-10; ↓iNOS, TNF-α	Indirect: TLR4 involvement inferred from inhibitor studies [78]; direct engagement not established; other receptors may contribute	Antibacterial; M2 polarisation; 94.5% infected wound closure	[78,79,80,81,82,83,84]
Mannose-free	HA	Injectable hydrogel; nanocomposite; patch	In vitro: macrophages [87]; In vivo: mouse diabetic [87], rat full-thickness [89]	↑CD206, Arg-1, IL-10; ↓TNF-α	Indirect: MW-dependent effects established in the foundational literature (not from [85]); CD44 established but not validated in wound studies; pathway inhibitors used [87]	Anti-inflammatory; granulation; collagen deposition	[87,89,90]
Mannose-free	SA	Composite hydrogel; composite hydrogel; aerogel; microsphere; microneedle	In vivo: mouse diabetic, rat full-thickness	Not directly polarising; markers examined with other actives	Not directly evaluated—alginate primarily carrier/scaffold; no independent M2-polarising activity reported	Exudate absorption; mechanical strength; bioactive cargo carrier	[91,92,93,94,95,96,97,98]
Mannose-free	Laminarin	Oral nanogene delivery (not skin)	In vivo: colitis (non-cutaneous; mechanistic) [101]	↑CD206, IL-10 (in colitis model)	Indirect for skin M2: Dectin-1 binding established for β-glucans [99]; Laminarin-specific M2 activity not skin-validated	Targeted polarisation in colitis; skin not reported	[99,100,101]
Mannose-free	LBP	Conductive polyphenol microneedle	In vivo: rat spinal cord injury (non-cutaneous mechanistic); mouse diabetic wound	Not specified in wound model	Indirect: M1/M2 phenotype shift observed in non-cutaneous model [103]; no receptor identified; LBP effect not isolated from electroacupuncture [104]	Diabetic wound healing; reduced depressive-like behaviours	[102,103,104]
Mannose-free	APS	Microneedle; 3D-printed scaffold	In vitro: macrophages; In vivo: mouse diabetic, rat full-thickness	↑CD206, IL-10; ↓iNOS, TNF-α	Indirect: β-catenin/NF-κB via inhibitor studies [106,108]; receptor not identified	M2 polarisation at late inflammation; angiogenesis	[106,107,108]
Mannose-free	PSP	Not specified (direct in vivo administration)	In vivo: mouse full-thickness wound	↑CD206, IL-10; ↓iNOS, TNF-α (scRNA-seq)	Indirect: PI3K/Akt inferred from scRNA-seq correlation only [109]; not inhibitor-validated	Collagen deposition; anti-inflammatory; M2 polarisation	[109]

## Data Availability

No new data were created or analyzed in this study. Data sharing is not applicable to this article.

## Abbreviations

The following abbreviations are used in this manuscript:

AceKGM	acetylated konjac glucomannan
ACS	aldehyde-modified chitosan
AGEs	advanced glycosylation end products
AHA	aldehyde-grafted sodium hyaluronate
AI	artificial intelligence
AL	astilbin liposomes
Alg-DA	dopamine-functionalized alginate
AP-CD	aloe polysaccharide–collagen composite dressing
APS	Astragalus polysaccharide
Arg	arginine
Arg-1	arginase-1
AST	astaxanthin
BA-ODex	BA-grafted oxidized dextran
BDDE	1,4-butanediol diglycidyl ether
BSP	Bletilla striata polysaccharide
CA	chlorogenic acid
CFCS	Catechol-functionalized chitosan
CMC	carboxymethyl cellulose
CMCS	carboxymethyl chitosan
CPP	chitosan-poloxamer polymer
CS	chitosan
CQHP	quaternized chitosan supramolecular hydrogel loaded with hesperidin
DA	dopamine
DA-CNFs	dopamine-functionalized cellulose nanofibers
DDA	degree of deacetylation
DOP	Dendrobium officinale polysaccharide
DS	diclofenac sodium
EA	electroacupuncture
ECM	extracellular matrix
ENM	electrospun nanofibrous membrane
G	α-L-guluronic acid
GCS	Guanidinium-functionalized chitosan
GFNPs	Ganoderma lucidum polysaccharide–ferulic acid nanoparticles
GLP	Ganoderma lucidum polysaccharide
GMP	Good Manufacturing Practice
HA	hyaluronic acid
HAMA	hyaluronic acid methacrylate
HBP	hexosamine biosynthesis pathway
HMW-HA	high-molecular-weight hyaluronic acid
iNOS	inducible nitric oxide synthase
KGM	konjac glucomannan
KMOS	konjac glucomannan oligosaccharides
LBP	Lycium barbarum polysaccharide
LINC	linker of nucleoskeleton and cytoskeleton
LMW-HA	low-molecular-weight hyaluronic acid
M	β-D-mannuronic acid
MMPs	matrix metalloproteinases
MR	mannose receptor
MRSA	methicillin-resistant Staphylococcus aureus
NO	nitric oxide
OAP	oxidized aloe polysaccharide
OSA	oxidized sodium alginate
oxi-CNC	aldehyde-modified cellulose nanocrystals
OGLP	oxidized Ganoderma lucidum polysaccharide
PAC	Periplaneta americana remnant chitosan
PA	Periplaneta americana remnant polysaccharide
PAPCF	Periplaneta americana remnant polysaccharide-chitosan composite film
PAAS	sodium polyacrylate
PBA	phenylboronic acid
PIL-NH_2_	amino-modified imidazole salts
PRP	Platelet-rich plasma
PSP	Hylocereus undatus stem polysaccharide
PVA	polyvinyl alcohol
Res	resveratrol
rhEGF	recombinant human epidermal growth factor
ROS	reactive oxygen species
SA	sodium alginate
SAR	Structure–activity relationship
SCgel	self-healing chitosan hydrogels
sHA-CA	catechol-grafted sulfated hyaluronan
TA	tannic acid
YSPs	yolk–shell structures
